# Tailored implementation of a behaviour change intervention for post-stroke physical activity: A mixed-methods feasibility study

**DOI:** 10.1177/02692155251382502

**Published:** 2025-10-03

**Authors:** Sarah A Moore, Jessica Calder, Sebastian Potthoff

**Affiliations:** 1 The School of Sport, Exercise and Rehabilitation, Health and Life Science, 5995 Northumbria University, Newcastle Upon Tyne, UK; 2 Stroke Research Group, Faculty of Medical Science, Newcastle University, Newcastle Upon Tyne, UK; 3 Research and Development, Northumbria Healthcare NHS Foundation Trust, North Shields, UK; 4 School of Communities and Education, Health and Life Science, Northumbria University, Newcastle Upon Tyne, UK

**Keywords:** Stroke, rehabilitation, physical activity, implementation, tailoring

## Abstract

**Objective:**

To explore the feasibility of tailored implementation of a stroke physical activity behaviour change intervention (Physical Activity Routines After Stroke (PARAS)) and the feasibility of the intervention.

**Design:**

Feasibility study applying mixed methods.

**Setting:**

UK stroke services.

**Participants:**

Four stroke rehabilitation teams comprising 34 healthcare professionals.

**Intervention:**

We applied a tailored implementation process informed by the Integrated Theory-based Framework for Intervention Tailoring Strategies to the PARAS intervention. Teams each attended two facilitated workshops (face-to-face/hybrid/online) identifying barriers and facilitators (determinants) to PARAS implementation to enable development of tailored implementation plans. Plans were applied and reviewed.

**Main measures:**

Feasibility of the tailored implementation process and intervention was explored via analysis of online questionnaire responses and thematic analysis of workshop and review session content. Inductive analysis identified determinants to implementation plan completion and enabled mapping of promising implementation strategies and their operationalisation and intervention development needs.

**Results:**

Thirty-four healthcare professionals participated across four teams. The facilitated, tailored implementation process was deemed feasible. All teams reported partially achieving implementation plans. Factors influencing implementation plan success included: motivation; stakeholder involvement; leadership and planning; intervention delivery skills. Implementation strategies mapped to factors included: assess for readiness; build a coalition; identify champions; train for leadership; and develop an implementation plan and ongoing training. Intervention adaptations identified included intervention tailoring and digitising resources.

**Conclusion:**

Our implementation process and the PARAS intervention were feasible with moderate amendments. Our findings enabled development of a model to support tailored implementation of PARAS and identified intervention development needs to guide future evaluation.

## Introduction

There are 1.3 million stroke survivors in the UK.^1^ Stroke can result in a range of physical, cognitive and perceptual impairments, reducing quality of life and increasing vulnerability to further health problems.^2^ Evidence supports physical activity as beneficial in managing many post-stroke challenges, and the UK National Clinical Guidelines for Stroke (2023) recommend personalised support to help stroke survivors be active.^3^

Despite evidence and guidelines, physical activity levels are low post-stroke.^4^ This reflects a broader issue with many evidence-based stroke rehabilitation interventions and guidelines failing to translate to practice.^5^ Part of this problem may be intervention design without consideration of future implementation needs within complex systems such as stroke rehabilitation.^5,6^ Indeed, the Medical Research Council Framework for developing and evaluating complex interventions recommends consideration of programme theory and implementation within early design and feasibility testing prior to large-scale evaluation.^7^

Implementation science studies how to integrate evidence into routine practice.^8^ Success in this area is more likely when guided by theory and evidence-based strategies.^9,10^ Normalisation Process Theory is one such theory, examining how interventions become embedded in practice through teamwork.^11^ Normalisation Process Theory identifies key factors that influence whether practices are adopted and sustained in healthcare.

Once these factors are understood, tailored strategies can be selected. The Expert Recommendations for Implementing Change (ERIC) taxonomy incudes 73 such strategies.^9^ This process known as ‘implementation tailoring’^12^ has shown effectiveness across healthcare settings.^10,13,14^ For example, in the ImpleMentALL study tailored implementation was found to be more effective than standard processes in embedding internet-based cognitive behavioural therapy.^13^ The Integrated Theory-based Framework for Intervention Tailoring Strategies (ItFits-toolkit) was created for the ImpleMentALL study, and this toolkit could be adapted to aid implementation of interventions into complex stroke rehabilitation pathways.

Physical Activity Routines After Stroke (PARAS) is a co-designed evidence-and theory-informed intervention developed to promote physical activity after stroke.^15,16^ Our early feasibility work found PARAS to be feasible and acceptable but identified intervention adaptations to enable effectiveness and indicated a more tailored process may be required to aid implementation.^15^

This study explores the feasibility of facilitated, tailored implementation applying the ItFits-toolkit with stroke teams delivering the PARAS intervention and further explores the feasibility of the intervention. Study findings will inform a future definitive hybrid trial evaluating the PARAS intervention and the tailored implementation process.

### Objectives

1. To explore the feasibility and costs of using facilitated, tailored implementation with stroke teams delivering PARAS.
2. To explore the outcome of using facilitated, tailored implementation to enable the implementation of PARAS.
3. To explore barriers and facilitators to teams achieving their PARAS-tailored implementation plans.
4. To develop a logic model and guide for future facilitated, tailored implementation of PARAS.
5. To explore the feasibility of the PARAS intervention.

## Methods

This study has been described following the Standards for Reporting Implementation Studies.^17^

*Design:* A tailored implementation and feasibility study applying mixed-methods was conducted. The study received ethical approval from Northumbria University on 09/02/24 Reference: Moore 2024-6625-6050.

*Participants:* UK Stroke services (National Health Service inpatient; early supported discharge; outpatients and domiciliary services *and* services provided in the community by council / commercial leisure services or third sector organisations) with a role supporting physical activity were invited to take part in the study.

The study was advertised by two methods: (1) email to PARAS website members and (2) email to teams who had previously received PARAS training from members of the research team. When an individual email was sent to a PARAS website member, they were asked if their team wished to be involved. We purposefully sampled individuals / teams with a potential interest in PARAS to enable the likelihood of implementation of PARAS, and we ensured our sample included users of PARAS and those without prior experience.

Eligible participants were provided with a participant information sheet and after sufficient time to consider this information (over 24 h) provided informed written consent.

### Intervention

We applied a tailored implementation process to support delivery of the PARAS intervention. A full intervention description applying the Template for Intervention Description and Replication can be found in Supplementary Materials Appendix A. In brief, PARAS is a behaviour change programme promoting physical activity among stroke survivors who are able to move more and have no contraindications. PARAS was co-developed with stroke survivors, carers and healthcare professionals and is grounded in behaviour change theory. It is facilitated by a stroke professional who supports the individual to monitor activity, review benefits, set goals, create action and coping plans and track progress. The intervention is supported by a workbook and tools such as diaries and goal sheets and is designed for use across the stroke pathway. All training and resources are freely available at https://paras-strokerehab.org/.

**Figure 1.**
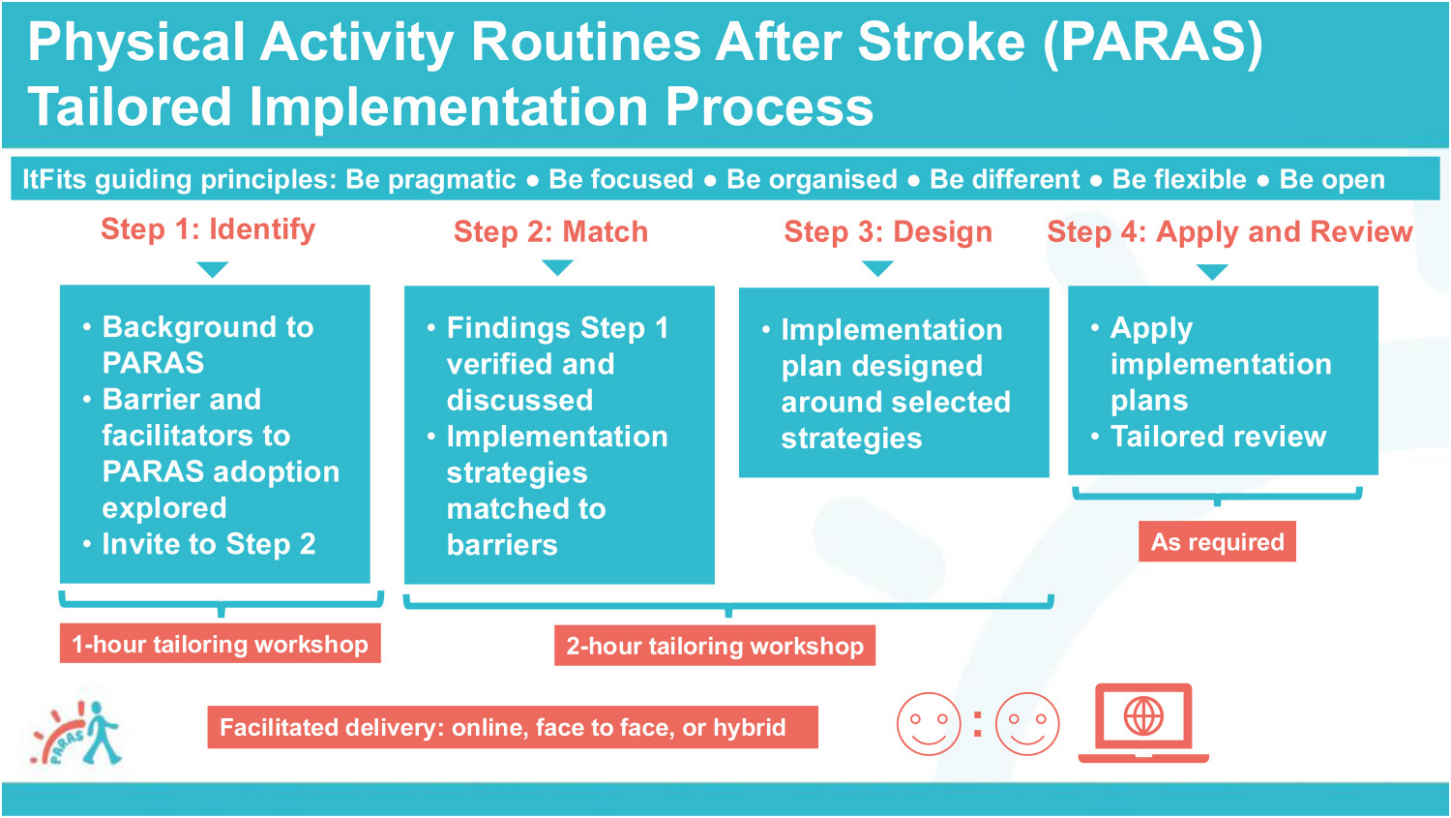
Physical Activity Routines After Stroke tailored implementation process.

The tailored implementation process was guided by the Integrated Theory-based Framework for Intervention Tailoring Strategies (ItFits-toolkit), which includes four core steps: (1) identify goals and barriers, (2) match barriers to strategies, (3) design a local plan and (4) apply strategies and review progress.^18^ Six guiding principles from The ItFits-toolkit – pragmatic, focused, organised, different, flexible and open – were adapted for PARAS. Use of The ItFits-toolkit has previously been self-guided; however, our process was facilitated by a research team comprising a clinical academic physiotherapist (SaM) and a research assistant (JC). Facilitation was offered face-to-face, hybrid or online (via Microsoft Teams), depending on location. Each step is described in detail below and summarised in Figure 1 with the guiding principles outlined in Table 1.

**Table 1.** The ItFits-toolkit principles tailored to Physical Routines After Stroke.

Principle	Tailoring to Physical Activity Routines After Stroke
**1. Be pragmatic**: *Focus on realistic achievable steps*	Daily delivery of stroke services is demanding with variable patient and service needs and constant shifts in staffing. The facilitator encouraged teams to focus on realistic steps within their setting.
**2. Be focused:** *Focus on one thing at a time don’t try to do everything at once*	The facilitator encouraged teams to identify specific goals and objectives prioritising key barriers.
**3. Be organised:** *Each step needs an identified owner to take responsibility for delivery*	Within the implementation plan teams were asked to consider who would be involved in the action plan.
**4. Be different:** *Do not focus on things you feel comfortable with or the things that you would normally do*	The facilitator encouraged teams to embrace new ways of working to enable PARAS delivery and consider how different implementation strategies may be tailored to individual areas.
**5. Be flexible:** T*he same solution may not work for everyone so be prepared to adapt your plans and ideas*	Teams were encouraged to tailor plans to settings with an awareness that although individuals worked in the same team, they may work in different parts of the stroke pathway / settings.
**6. Be open:** L*isten to and value your stakeholders’ knowledge and experience*	The facilitator enabled a safe space where team members could be heard and have their opinions valued.

*Step 1: Identify:* The first one-hour workshop introduced the PARAS intervention and explored barriers and facilitators to implementation through semi-structured questions, informed by Normalisation Process Theory. These covered awareness, initiation, experience and integration of PARAS (Supplementary Materials Appendix B). At the end of the session, teams were asked if they wished to continue and, if so, a second workshop was scheduled.

*Step 2 and 3: Match and design:* The second workshop (2 h) focused on verifying and discussing identified barriers and matching them with strategies. Teams were supported in developing an implementation plan including goals, strategies, action and coping plans and success measures. Following challenges with the first workshop, we revised the implementation template to include structured sections and examples aligned with the PARAS goal-setting process, helping to reinforce behavioural techniques (Supplementary Materials Appendix C V1).

To support strategy selection, teams received a refined list of 26 strategies adapted from the original 73-item ERIC compilation.^9^ Strategies were grouped (e.g., planning, educational) for easier navigation. Early workshops used paper copies, which proved cumbersome, so a synthesised strategy table (Supplementary Materials Appendix D) was used in later sessions. Resources such as the summary of barriers, the plan template and the strategies table were shared ahead of time for efficiency.

Each team completed a paper implementation plan, which was digitised and shared back. If unfinished, teams submitted digital versions afterward.

*Step 4: Apply and review:* Teams implemented their plans with their preferred level of support – via online meetings, face-to-face sessions (where possible) or email. Teams chose their own timelines based on service needs and preferences for review.

### Data collection

*Demographics:* Team and individual characteristics were collected at the start of the first workshop, with updates for new members. Data included service structure, sex, age range, profession, NHS banding, years working in stroke, main area of work, relevant training and PARAS status (adopter, potential adopter or non-adopter).

*Observations:* Workshops and review sessions were audio-recorded (Dictaphone), video-recorded (Microsoft Teams) and accompanied by field notes (taken by JC or SP). Recordings were transcribed and checked for accuracy. Relevant email communications and digital implementation plans were also archived.

*Questionnaires:* A feasibility and an outcomes questionnaire were developed for the study and circulated using Microsoft Outlook 365 Forms. The feasibility questionnaire had five Likert-scale questions exploring acceptability of each step with the option to comment further and two open questions on what they liked about the process and what could be improved. It was administered after completion of Steps 1-3. The outcomes questionnaire had open questions exploring plan achievement, perceived barriers/facilitators, strategy effectiveness and examples of success and was administered after Step 4. Some teams opted to answer the outcomes questionnaire over a Teams meeting.

*Costs:* Travel, subsistence and research staff costs were recorded.

### Data analysis

Team characteristics, closed survey responses and implementation plans were summarised descriptively. Open survey questions were thematically analysed.

To establish if the tailored implementation process and PARAS intervention were feasible, we applied the traffic light system described by Avery et al.^19^ If only minor amendments to the implementation process (e.g., small amount extra time for delivery) and intervention (e.g., minor changes to enable intervention tailoring) were required and costs were low (<£100) this was classified green, moderate amendments (e.g., moderate changes to implementation process or intervention content adaptations required) and costs <£500 were classified amber and if significant amendments (e.g., large changes to process or intervention content) and costs (>£500) were identified this was classified red. It would only be appropriate to progress to the next stage of evaluation if the feasibility was classified green or amber.

To explore implementation barriers, enablers and potential PARAS intervention development needs, qualitative data from workshops, reviews, questionnaires and emails were analysed inductively, drawing on principles from first- and third-generation Grounded Theory.^20^ The analysis was undertaken by SaM (a research physiotherapist and member of PARAS development team) and JC (a research assistant with no prior knowledge of PARAS) with any disputes discussed with SP (a health psychologist/implementation scientist with no prior knowledge of PARAS) with data coded and analysed by hand, that is, no qualitative software employed. For each team, data were chronologically ordered and line-by-line coded. Focused coding across teams helped identify central codes, from which categories and sub-categories (key determinants of practice) were developed.

These categories informed the mapping of suitable implementation strategies and methods to operationalise them. Analytical memos, team discussions and visual diagrams supported interpretation.

The findings contributed to the creation of a logic model and structured guide to support future delivery of the tailored implementation process. A logic model graphically depicts shared relationships of different mechanisms (what?) and processes (how?) within a study and are useful for summarising findings.^21^

### Sample size

This study did not include a sample size calculation due to the study design/aim and constraints of the project funding and timeline.

## Results

Four teams participated: three from the North East of England (previously trained in PARAS) and one from the South (recruited via PARAS website membership). A fifth North East team joined but only completed Step 1 due to project funding and timeline. The data from Step 1, including this fifth team, will be reported elsewhere. Additionally, a UK stroke charity for young survivors expressed interest, but with only one attendee, full team tailoring was not possible. Their input will inform future PARAS intervention development.

In total, 34 healthcare professionals took part across the four teams. Table 2 shows participant characteristics and delivery modes. Some individuals in Teams 2 and 3 had also been involved in the earlier PARAS feasibility study. Variations in attendance were due to staff changes, illness or team decisions to focus on specific services (e.g., inpatient vs. community). For instance, Team 2 targeted inpatient and early discharge, while Team 3 limited participation to community staff due to implementation challenges in inpatient settings.

**Table 2.** Team characteristics.

	Team 1	Team 2	Team 3	Team 4
Participants	Step 1 *n* = 6 Step 2 & 3 *n* = 4 (2 new)	Step 1 *n* = 8 Step 2 & 3 *n* = 4	Step 1 *n* = 6 Step 2 & 3 *n* = 2	Step 1 *n* = 8 Step 2 & 3 *n* = 12 (4 new)
Professions	3×Occupational therapists 1×Occupational therapy assistant 4×Physiotherapists	7×Physiotherapists 1×Physiotherapy assistant	5×Physiotherapists 1×student Physiotherapist	6×Physiotherapists 4×Occupational therapists 2×Rehabilitation assistants
Years of experience in stroke	<5 *n* = 5 5–10 *n* = 1 >10 *n* = 2	<5 *n* = 1 5–10 *n* = 2 >10 *n* = 5	<5 *n* = 1 5–10 *n* = 2 >10 *n* = 3	<5 *n* = 2 5–10 *n* = 3 >10 *n* = 7
Physical Activity Routines After Stroke adoption	Potential adopters *n* = 8	Adopter *n* = 1 Potential adopters *n* = 7	Adopter *n* = 1 Potential adopters *n* = 5	Potential adopters *n* = 12
Setting	Inpatient stroke *n* = 6 Community stroke *n* = 2	Inpatient stroke *n* = 3 Early Supported Discharge Team *n* = 2 General neurology outpatients / community *n* = 3	Inpatient stroke *n* = 4 Community stroke *n* = 2	General neurology outpatient / community *n* = 12
Mode of delivery Steps 1–3	Face-to-face	Hybrid	Face-to-face	Online
Mode of delivery review	Email	Online 2 × 30 min	Monthly email contact	Online review 2 × 60mins

### Objective 1: Feasibility and cost of facilitated, tailored implementation

An online questionnaire was used to explore the feasibility of the tailored implementation. Twelve healthcare professionals across the teams completed the questionnaire. Responses to the Likert scales indicated that most participants found the tailored implementation process useful and helpful (Table 3).

**Table 3.** Responses to Likert questions embedded within feasibility questionnaire.

	Strongly agree	Agree	Neutral	Disagree	Strongly disagree
Questions	Number (percentage)				
*Presenting intervention background was useful at the start of the workshop.*	5 (42)	6 (50)	1 (8)	0	0
*The initial workshop helped to identify barriers and facilitators to implementing intervention*	5 (42)	6 (50)	1 (8)	0	0
*Summarising barriers and facilitators identified in the workshop was a helpful reminder*	6 (50%)	5 (42%)	1 (8)	0	0
*Working through the implementation plan was helpful*	4 (33)	8 (67)	0	0	0
*The table with examples of implementation strategies was useful*	3 (25)	7 (58)	1 (8)	0	0
*It was useful to think about outcomes of the implementation plan*	3 (25)	9 (75)	0	0	0

Analysis of open question responses

The teams valued having a facilitator to guide the implementation process and to provide reminders. This finding was supported by data from the workshops.

‘…Little bit of a nudge how you're getting on [from the facilitator] probably does help.’ (Occupational therapist, workshop)

Sharing of ideas and generating plans as a team was the part of the implementation process most valued by the healthcare professionals. Having time away from practice to focus and develop plans was viewed as helpful:

‘Good to have time as a team to take a step back from clinical practice and spend time developing plan in the meeting.’ (anonymous questionnaire response)

The healthcare professionals found the steps in the implementation process logical and structured and enjoyed the practical and interactive elements. Sharing ideas and feedback on PARAS from patients helped to shape implementation plans.

‘Interactive - a good way of coming up with ideas and seeking others’ opinions on these. Nice to hear feedback from patients.’ (anonymous questionnaire response)

One healthcare professional, however, found it hard to attend the workshop due to clinical pressures, and another commented it was difficult to regularly implement PARAS due to clinical demands. Most healthcare professionals did not have any suggestions for improvements to the implementation process. The only improvements suggested were: examples of how other teams had implemented PARAS (*n* = 2 healthcare professionals); examples of use with patients (*n* = 1 × healthcare professional); more training on PARAS (*n* = 2 × healthcare professionals) and inviting more team members to make implementation easier (*n* = 1 × healthcare professional). These suggestions link with themes described below in the outcomes section.

With regards to mode of delivery of the implementation process most respondents reported online worked well, was easier than face-to-face and aided inclusion. Two respondents preferred face-to-face.

The costs of delivering the facilitated, tailored implementation can be viewed in Table 4. Online delivery was cheaper without the need for travel costs and refreshments. Online and face-to-face delivery costs per team were less than £500. We included costs for transcription between Steps 1 and 2, but do not envisage this cost would be required in future iterations of the implementation process. Overall delivery and costs of tailored implementation were deemed feasible according to our criteria.

**Table 4.** Delivery costs of facilitated, tailored implementation process.

Item	Average cost per team face-to-face delivery (£)	Cost of online delivery (£)	Total across project (£)
Research team travel	43.87	0	131.62
Refreshments	6.40	0	19.60
Transcription costs between workshop 1 and 2	92	92	368
Workshop resources	32.50 (workbooks and photocopies)	40 (workbooks and delivery)	137.50
Lead researcher and research assistant costs to prepare and deliver facilitation of steps 1–4	344.93 (including costs travel time)	258.70	1293.50
Total	519.7	390.7	1950.22

### Objective 2: Outcome of using tailored implementation

Outcome of the tailored implementation was explored through analysis of the content of the implementation plans and review of self-reported achievement and impact of plans via outcome questionnaire results.

Two teams designed one implementation plan, the third team had one plan for inpatients and another for community and the final team split their plan into two areas of focus: training and delivery. Examples of implementation plans can be viewed in Supplementary Materials Appendix E All four teams reported they had partially achieved their implementation plans.

*Team 1* created separate plans for inpatient and community settings. The community team reported use of PARAS with five patients, noting improvements in confidence and goal achievement. The inpatient team did not implement PARAS due to staffing shortages and competing priorities.

*Team 2* spanning two sites, aimed to use PARAS with six patients in three months. They reported partial success, with greater progress at one site. A staff rotation disrupted continuity.

*Team 3* initially included inpatient and community staff, but staffing issues led them to focus on community only. The plan was not achieved within the time frame. Notably, inpatient staff who didn’t attend later workshops still advanced implementation independently, embedding PARAS into a six-month quality improvement project and ensuring new staff completed PARAS website training.

*Team 4* had the most success. Their first goal – to have all staff complete PARAS website training – was fully met. Their second goal, to apply PARAS with 1–2 patients each, was reported to be 90% achieved, limited only by the small number of suitable stroke patients in their general neurology caseload.

### Objective 3: Barriers and facilitators to achievement of implementation plans

Barriers and facilitators to implementation plan success were explored through qualitative analysis of workshop, review and email content. Categories and sub-categories that emerged from the data can be viewed in Table 5 and are described below. Exemplar quotes for each category can be viewed in Supplementary Materials Appendix F.

**Table 5.** Mapping of implementation strategies to categories and sub-categories identified relating to barriers and facilitators to team implementation plans for Physical Activity Routines After Stroke.

Category	Sub-category	Implementation strategy defined by Expert Recommendations for Implementing Change compilation^9^
Motivation to implement Physical Activity Routines After Stroke	When Physical Activity Routines After Stroke aligns with team priorities / philosophy / skills implementation is more likely	Assess for readiness and identify barriers and facilitators
	Sharing Physical Activity Routines After Stroke success stories aids motivation to implement Physical Activity Routines After Stroke	Obtain and use patients/consumers and family feedback
Stakeholder involvement in Physical Activity Routines After Stroke implementation	Core team engagement enables Physical Activity Routines After Stroke implementation	Build a coalition
	Multi-professional team awareness of Physical Activity Routines After Stroke aids implementation	Build a coalition
Leadership and planning of Physical Activity Routines After Stroke implementation	Implementation facilitators support implementation	Facilitation
	Identifying Physical Activity Routines After Stroke champions enables implementation success	Identify and prepare champions
	Forming clear tailored implementation plans aids implementation	Develop a formal implementation blueprint
	Implementation plan reminders / prompts enable implementation	Remind clinicians & Change record systems
	Having suitable Physical Activity Routines After Stroke resources available enables implementation	Change physical structure and equipment
Physical Activity Routines After Stroke delivery skill acquisition	Training and use of Physical Activity Routines After Stroke can enable implementation and fidelity	Conduct ongoing training
	Training and making rotational staff champions can aid implementation	Recruit, designate and train for leadership
	Becoming familiar with Physical Activity Routines After Stroke aids self-efficacy and ability to adapt delivery	Stage implementation scale-up
	Peer support and clinical supervision aids Physical Activity Routines After Stroke implementation	Organise clinician implementation team meetings & Identify early adopters

Motivation emerged as an important driver to implementation plan success. One team stated they had a ‘philosophy of behaviour change’ and had previous training and experience in delivering person-centred goal setting and motivational interviewing. This aided their motivation to implement PARAS as the team perceived the content of the intervention aligned with their service culture and delivery methods. This team understood the techniques embedded within PARAS aiding their confidence in delivery:

‘…I think a lot of the content and the delivery was quite familiar. So we were quite comfortable with a lot of the language that was used around sort of barriers, facilitators, goals’ (Team 4, Physiotherapist, review)

This team were also positive about the PARAS intervention concept of ‘move more, sit less’ physical activity rather than ‘exercise’. The broad concept of physical activity was reported to be more accessible to stroke survivors, providing more opportunities to undertake physical activity and a different way to think about activities that fall under the spectrum of physical activity:

‘…I do think that having that variety of it wasn't just exercise, it was very much activity… it made them think about it in a different way.’ (Team 4, Physiotherapist, review)

A barrier to motivation to achieve implementation plans was prioritisation. When PARAS was not considered a priority plans were often not completed. This was confounded when staff left or rotated out of services reducing time and motivation to deliver initial plans:

‘…We just haven't had the capacity to look at this since our last meeting. It's on my list of things to do once things quieten down a bit.’ (Team 1, Occupational therapist, email)

Seeing the positive effects of PARAS motivated implementation, while sharing successes helped teams identify potential recipients. When feedback mechanisms were absent, this was viewed as a barrier, with one team citing it as a reason for not completing their plans:

‘…I think certainly something from this that we haven't really done is kind of getting that feedback… if you know if someone was to do the training and then utilize that, getting the feedback from the patient, but also the feedback from the therapist on how they found that.’ (Team 3, Physiotherapist, review)

Implementation was more successful when delivery was a shared team process rather than dependent on individual ‘champions’, making it more manageable. In one team, responsibility fell mainly on a designated PARAS adopter and a rotational staff member; when the latter left, delivery was disrupted:

‘…at the minute it is just completely reliant upon certain individuals being around and then being free enough to kind of take the time to deliver.’ (Team 2, Physiotherapist, review)

Increased success with implementation plans was observed when as many relevant stakeholders were engaged as possible initially and during the process. When key stakeholders were not included it reduced the critical mass of individuals to drive plans forward:

‘…I know it would be a massive extra bit of work, but I don't know whether or not to kind of get [Team name who were not involved at start of implementation process] involved.’ (Team 2, Physiotherapist, review)

Awareness of implementation plans and knowledge of PARAS across the wider multidisciplinary team were seen as key to success. Implementation could influence stroke care in other areas, with certain members, such as speech and language therapists, facilitating delivery for patients with aphasia:

‘…I think it's about educating everybody though, isn’t it? It's about introducing it to not just the physios but obviously the OTs as well and even the speech and language therapists’ (Team 1, Physiotherapist, workshop)

Aligned with questionnaire findings, teams saw the value of an external facilitator to organise meetings and provide reminders, which were especially useful during busy periods. Teams also highlighted the importance of internal reminder systems to keep implementation from slipping ‘off the radar’. Sharing progress and successes alongside weekly prompts was seen to enhance motivation and plan enactment:

‘…just a weekly reminder of who's starting it, with who and how it's going.’ (Band 6, Physiotherapist, review)

Embedding these reminders within current systems was suggested as a potential way to enable the normalisation of the implementation of PARAS:

‘…we do have a bit of like an MDT sheet that we go through and we're looking at kind of outcome measures. And I just wonder if that could be kind of added on as a consideration.’ (Team 3, Physiotherapist, review)

The importance of a champion was regularly linked with implementation success or failure. Champions were perceived to be very ‘helpful’ as an aid to ‘prompt, remind and encourage’ staff through the process and as an important source of knowledge. When champions were not clearly defined, or the leadership role was not undertaken successfully, this was associated with implementation plan failure:

‘…I think ultimately I genuinely haven't kind of pushed this forward probably strongly enough…. I haven't. Kind of enforced that with my staff, so I definitely feel that I haven't led on that very well.’ (Team 3, Physiotherapist, review)

As noted in the stakeholder involvement category, champions were only effective when a critical mass of team members were engaged and clear leadership roles established. Such roles relied on detailed implementation plans with ‘logical’ actions and assigned tasks; where these were absent, as in some teams, success was compromised:

‘…I think firstly, I should have got this written up immediately and had it as more of a priority…. I wonder if you know a plan was there earlier whether that might have changed things.’ (Team 3, Physiotherapist, review)

Building implementation plans into local audit processes was discussed as a method of enabling implementation plans and measuring outcomes.

A common barrier arose when PARAS resources were unavailable, such as attending community visits without a workbook. Organising the environment to ensure resources were accessible increased implementation success:

‘…We've got some booklets that were in like, each of the places, so some of those, I'm sure there's more we could do to make it practically easier to use it, but that's sort of where we started. (Team 4, Physiotherapist, review)

Beyond availability, the suitability of PARAS resources was also identified as a determinant of practice. As patients and carers currently handwrite goals in the workbook, the lack of digital options limits integration with electronic medical records. One team addressed this by creating editable documents:

‘…she went on and saved the documents into a folder, made them editable for us so that we could email.’ (Team 4, Physiotherapist, review)

Only one team embedded PARAS website training into their implementation plan. Other teams built skills through practice or peer support from experienced staff. Limited initial knowledge was seen as a barrier to competence and confidence, and in some teams only a few individuals were motivated and confident to deliver the intervention:

‘…Getting that member of staff who's keen to do it, but then making sure that they're in a position where they're comfortable working with, instruct to be able to do this and they're making sort of good decisions around which patients are using it and which ones aren't.’ (Team 2, Physiotherapist, review)

The team that used the PARAS website training operated across two sites and appointed a champion in each. Training formats differed – one team completed it in a block, the other in smaller chunks – yet both were seen to work ‘quite well’. Peer support was also valuable: in one case, a PARAS adopter supported a rotational staff member to deliver the intervention, highlighting the importance of training rotational staff for sustainability. In another team, healthcare professionals shared delivery experiences, which aided reflection, learning and skill development:

‘…a lot of it seems to have developed over these sharing of a patient or discussing it after a patient or just reflecting in conversation, doing the training with someone else or alongside your team maybe.’ (Team 4, Physiotherapist, review)

Familiarity with PARAS influenced healthcare professionals’ self-efficacy and ability to adapt delivery for complex patients. Less experienced staff tended to select patients they perceived as more suitable or straightforward, while experienced staff adapted delivery to individual needs. Selecting patients more likely to succeed appeared to facilitate implementation by building confidence and self-efficacy.

‘I think that's why I chose these certain people as well because. I wasn’t familiar with it, so that was a good opportunity to kind of try it’. (Team 1, Occupational therapist, workshop)

### Objective 4: Development of a logic model and guide for future tailored implementation of PARAS

**Figure 2.**
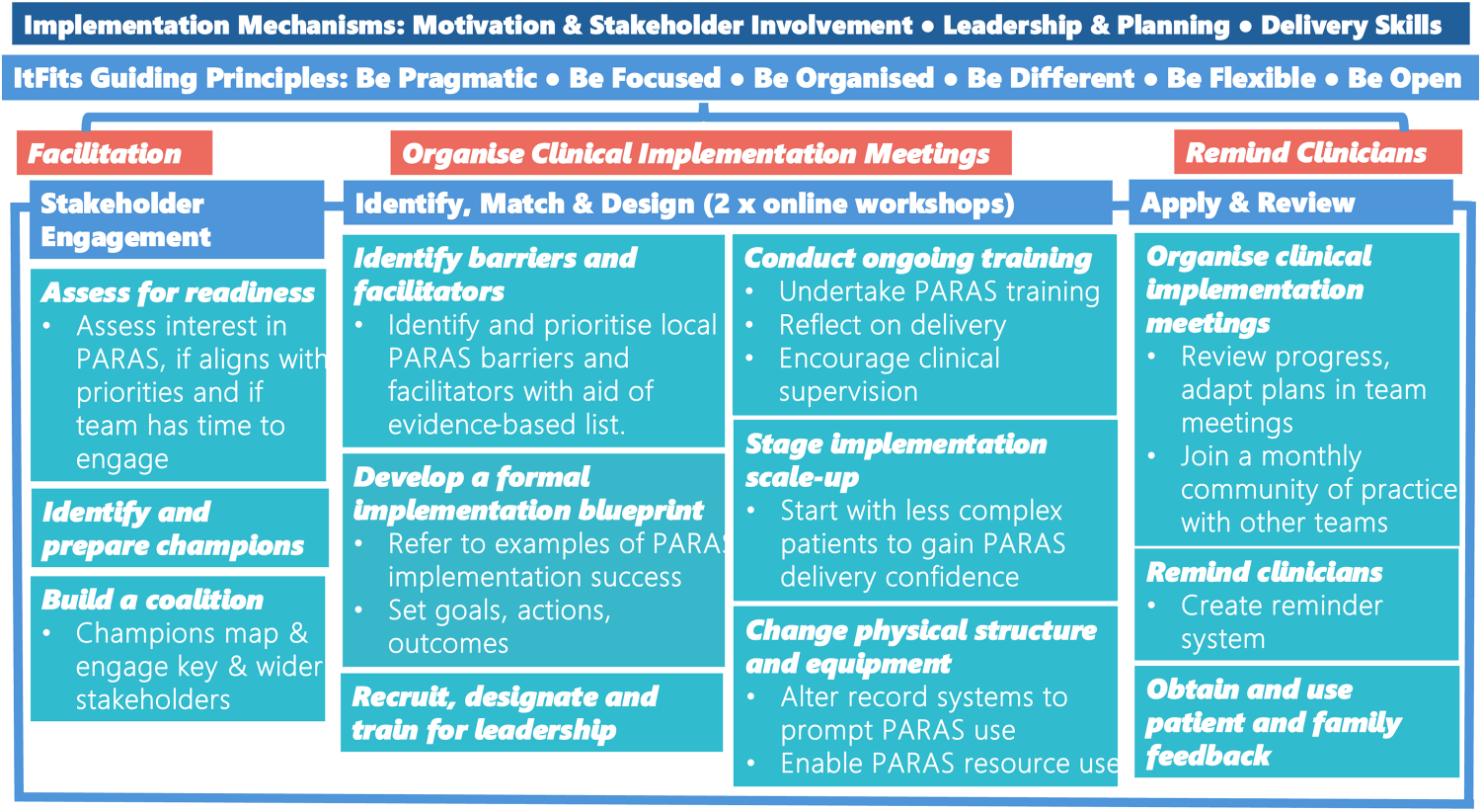
Logic model for future facilitated, tailored implementation of Physical Activity Routines After Stroke. The implementation mechanisms that emerged from this research overarch the model (dark blue). The Integrated Theory-based Framework for Intervention Tailoring Strategies guiding principles and stages form the framework for the process (sky blue). An external facilitator guides the stages and embedding of the principles (orange). Stakeholder engagement is considered in detail at the start and throughout the process. Within each of the stages, implementation strategies (text in italics) that were perceived as promising are applied by the facilitator and the healthcare professionals.

Barriers and enablers to implementation plans were used to inform the selection of implementation strategies (defined by the ERIC compilation viewed in Table 5) which were embedded into a logic model for future delivery of tailored implementation process of PARAS (Figure 2). A comprehensive overview of the framework of the logic model and rationale can be viewed in Supplementary Materials Appendix G.

### Objective 5: PARAS intervention feasibility

Qualitative analysis identified moderate adaptations required to the intervention to enable implementation indicating the intervention is feasible. Adaptations alongside exemplar quotes can be viewed in Supplementary Materials Appendix H.

Adaptations to PARAS resources included the ability to use either the whole workbook or to have the option to select individual parts of the workbook to enable tailoring and ‘personalisation’ of delivery. Providing the option of different age-appropriate versions was also suggested:

‘…so like a personalised pack rather than just having that one booklet, where you can build your own booklet for them, depending on what they need.’ (Physiotherapist, Focus group)

Healthcare professionals raised concerns about some outcome measures, particularly the International Physical Activity Questionnaire,^22^ noting challenges with patient recall and scoring. While useful for discussing inactivity, some suggested adding a fatigue diary to complement physical activity diaries, as fatigue was often discussed in sessions. Although the ‘sit less, move more’ concept was valued, patients were thought to need clearer explanations of what counts as physical activity. As noted earlier, the handwritten-only workbook was also seen as a limitation, with calls for a digital version to support record keeping:

‘…So it did feel….a little bit clunky in terms of that documentation. Or quite or a bit more time consuming. So having some sort of booklet online that they could type directly into and e-mail it to you or you could look at it together.’ (Occupational therapist, review)

Healthcare professionals suggested developing new resources to broaden PARAS delivery. Although designed for one-to-one use, they saw potential for group formats and adaptation to other neurological conditions with minimal tailoring. They also identified a need for bespoke inpatient resources, as existing materials were developed for community use and had not been tested in inpatient settings, where implementation plans were less successful:

‘…probably less to do might be one that would be more use for the ward and maybe just a bit more ward specific, so maybe not as much information because they’re not going to have as much ability when they’re on a ward situation.’ (Physiotherapist, focus group)

## Discussion

This study explored the feasibility of delivering facilitated, tailored implementation of the PARAS intervention and the PARAS intervention. Both the process and the intervention were found to be feasible with moderate adaptation and research costs of facilitation were relatively low, indicating potential to progress to the next stage of evaluation. Findings are discussed considering existing literature on tailored implementation in healthcare and stroke rehabilitation.

Tailored implementation that targets specific determinants of practice is feasible, as supported by previous studies,^13,14^ and our results align. A notable benefit highlighted by teams was the chance to step back from clinical work to collaboratively address implementation challenges – echoing research on the importance of protected time.^13^

The facilitated implementation process, based on the ItFits-toolkit,^18^ was well received. Facilitators provided structure and encouraged reflection. Though the framework is usually self-guided, evidence from the ImpleMentALL study suggests that facilitation can enhance engagement.^13^ In stroke care, facilitators are recognised as key enablers of change.^23^

Clarifying the facilitator's role is important. Implementation science literature refers to facilitators as implementation support practitioners. The importance of this role is gaining support across the field.^24^ While facilitation adds cost, our economic analysis suggests this may be modest, and facilitation could be cost-effective if it boosts implementation success. One factor not considered was the cost of healthcare professional time required for implementation. These costs should be evaluated in future studies.

Stakeholder engagement emerged as a central factor in implementation, consistent with other stroke research.^25–29^ Limited early involvement from key stakeholders in some teams may have hindered progress. The fact implementation plans set were relatively unambitious may have been due to this lack of engagement as plan delivery was reliant on only a few individuals and teams were aware of further staffing disruption. For PARAS to have impact, broad engagement is essential, along with strategies to maintain implementation plans when staff rotate or leave. Such organisational barriers are common in stroke rehabilitation studies.^30^ The evaluation of the ImpleMentALL trial led to the development of the Implementation STakeholder Engagement Model (I-STEM),^31^ application of this model could guide and strengthen future PARAS implementation planning.

Stakeholder motivation was another key factor. Two teams had members from the original feasibility study,^15^ who may have felt obliged to participate. By contrast, a team recruited via the PARAS website was more successful – possibly because the intervention matched their values and skills. Better understanding and targeting of motivation may further support implementation,^32^ alongside strategies to embed motivation into routine practice.^33^ While reminders from facilitators or champions were useful, sustaining implementation without continual prompting remains challenging.

Training also played a role. Despite an available online intervention training package, only one team accessed it. Lack of training was highlighted by participants as a barrier to implementation, indicating training should be included in implementation plans. While training alone is insufficient,^34^ combining it with opportunities to practise and receive peer feedback was seen to build competence – aligned with evidence supporting clinical supervision.^35^

Teams reported difficulty using the ERIC compilation to select strategies.^9^ Even after reducing the original 73 strategies to a manageable subset, the process was still seen as complex. Prior studies have noted problems with strategy clarity and overlap.^36–39^ Simpler alternatives, such as the Behaviour Change Wheel's nine intervention functions, may be more accessible.^40^

Peer examples and support were also requested, echoing the ImpleMentALL trial findings that evidence-informed materials help practitioners select barriers and strategies.^13^ Our study will inform similar resources for PARAS implementation.

A limitation of our tailored process was the lack of structured guidance during the ‘apply and review’ stage. The ItFits-toolkit includes criteria and a timeline for reviewing plans,^18^ but teams created their own, sometimes loosely defined, schedules. Those with clearer timelines appeared more successful, suggesting that future iterations should offer structured review points and potentially include communities of practice to support progress.^18^

Identifying champions also helped implementation, consistent with existing research.^41^ Teams spread across locations benefitted from multiple champions to tailor support locally. However, depending on a few individuals’ risks sustainability,^42^ and broader leadership distribution is advised – as reflected in the Getting It Right First Time Stroke report.^43^

Implementation appeared more successful in community rather than inpatient settings. Although the intervention was adapted for inpatient use based on early feedback, our current findings suggest further modification may be required. Inpatient teams indicated their focus, and measurement tends to be on function and discharge preparation. Environmental and resource constraints may also affect implementation in inpatient care. In contrast, community settings can allow more personalised goal setting, broader activity choice and tools such as the International Physical Activity questionnaire^22^ and Warwick and Edinburgh Mental Well-Being Scale^44^ are more relevant.

Digital transformation was identified as another development area. The original workbook predated widespread use of electronic records, but digitisation aligns with current NHS priorities.^45^ Teams also expressed interest in adapting the intervention for other neurological conditions.

To conclude, our study demonstrates both the tailored implementation process and the PARAS intervention are feasible with moderate adaptation. Adaptations to the implementation process include improved stakeholder motivation and engagement, ensuring clear leadership and planning and increased focus on initial training in intervention delivery. Intervention adaptions to enable implementation include the ability to tailor to different context, adaptation of resources for inpatients and digitisation of workbooks.

Our findings will inform the iterative development of the PARAS intervention and our tailored implementation process for evaluation in a future definitive hybrid clinical effectiveness trial.

**Clinical messages**

- Implementation of complex interventions can be aided by early feasibility testing of both the intervention and implementation processes to guide development before full-scale evaluation.
- Tailored implementation is when local barriers and facilitators likely to influence intervention implementation are identified and strategies selected and applied to address them.
- We demonstrated the feasibility of using facilitated, tailored implementation in stroke rehabilitation to aid implementation of a physical activity behaviour change intervention (PARAS).
- Mechanisms influencing implementation of the behaviour change intervention included motivation and stakeholder involvement, leadership and planning and delivery skills. Intervention development needs identified to aid future implementation included tailoring for different contexts and resource digitisation.

## Supplemental Material

sj-pdf-1-cre-10.1177_02692155251382502 - Supplemental material for Tailored implementation of a behaviour change intervention for post-stroke physical activity: A mixed-methods feasibility study Supplemental material, sj-pdf-1-cre-10.1177_02692155251382502 for Tailored implementation of a behaviour change intervention for post-stroke physical activity: A mixed-methods feasibility study by Sarah A Moore, Jessica Calder and Sebastian Potthoff in Clinical Rehabilitation

sj-docx-2-cre-10.1177_02692155251382502 - Supplemental material for Tailored implementation of a behaviour change intervention for post-stroke physical activity: A mixed-methods feasibility study Supplemental material, sj-docx-2-cre-10.1177_02692155251382502 for Tailored implementation of a behaviour change intervention for post-stroke physical activity: A mixed-methods feasibility study by Sarah A Moore, Jessica Calder and Sebastian Potthoff in Clinical Rehabilitation

sj-docx-3-cre-10.1177_02692155251382502 - Supplemental material for Tailored implementation of a behaviour change intervention for post-stroke physical activity: A mixed-methods feasibility study Supplemental material, sj-docx-3-cre-10.1177_02692155251382502 for Tailored implementation of a behaviour change intervention for post-stroke physical activity: A mixed-methods feasibility study by Sarah A Moore, Jessica Calder and Sebastian Potthoff in Clinical Rehabilitation

sj-docx-4-cre-10.1177_02692155251382502 - Supplemental material for Tailored implementation of a behaviour change intervention for post-stroke physical activity: A mixed-methods feasibility study Supplemental material, sj-docx-4-cre-10.1177_02692155251382502 for Tailored implementation of a behaviour change intervention for post-stroke physical activity: A mixed-methods feasibility study by Sarah A Moore, Jessica Calder and Sebastian Potthoff in Clinical Rehabilitation

sj-docx-5-cre-10.1177_02692155251382502 - Supplemental material for Tailored implementation of a behaviour change intervention for post-stroke physical activity: A mixed-methods feasibility study Supplemental material, sj-docx-5-cre-10.1177_02692155251382502 for Tailored implementation of a behaviour change intervention for post-stroke physical activity: A mixed-methods feasibility study by Sarah A Moore, Jessica Calder and Sebastian Potthoff in Clinical Rehabilitation

sj-docx-6-cre-10.1177_02692155251382502 - Supplemental material for Tailored implementation of a behaviour change intervention for post-stroke physical activity: A mixed-methods feasibility study Supplemental material, sj-docx-6-cre-10.1177_02692155251382502 for Tailored implementation of a behaviour change intervention for post-stroke physical activity: A mixed-methods feasibility study by Sarah A Moore, Jessica Calder and Sebastian Potthoff in Clinical Rehabilitation

sj-docx-7-cre-10.1177_02692155251382502 - Supplemental material for Tailored implementation of a behaviour change intervention for post-stroke physical activity: A mixed-methods feasibility study Supplemental material, sj-docx-7-cre-10.1177_02692155251382502 for Tailored implementation of a behaviour change intervention for post-stroke physical activity: A mixed-methods feasibility study by Sarah A Moore, Jessica Calder and Sebastian Potthoff in Clinical Rehabilitation

sj-docx-8-cre-10.1177_02692155251382502 - Supplemental material for Tailored implementation of a behaviour change intervention for post-stroke physical activity: A mixed-methods feasibility study Supplemental material, sj-docx-8-cre-10.1177_02692155251382502 for Tailored implementation of a behaviour change intervention for post-stroke physical activity: A mixed-methods feasibility study by Sarah A Moore, Jessica Calder and Sebastian Potthoff in Clinical Rehabilitation

sj-docx-9-cre-10.1177_02692155251382502 - Supplemental material for Tailored implementation of a behaviour change intervention for post-stroke physical activity: A mixed-methods feasibility study Supplemental material, sj-docx-9-cre-10.1177_02692155251382502 for Tailored implementation of a behaviour change intervention for post-stroke physical activity: A mixed-methods feasibility study by Sarah A Moore, Jessica Calder and Sebastian Potthoff in Clinical Rehabilitation

sj-docx-10-cre-10.1177_02692155251382502 - Supplemental material for Tailored implementation of a behaviour change intervention for post-stroke physical activity: A mixed-methods feasibility study Supplemental material, sj-docx-10-cre-10.1177_02692155251382502 for Tailored implementation of a behaviour change intervention for post-stroke physical activity: A mixed-methods feasibility study by Sarah A Moore, Jessica Calder and Sebastian Potthoff in Clinical Rehabilitation

sj-docx-11-cre-10.1177_02692155251382502 - Supplemental material for Tailored implementation of a behaviour change intervention for post-stroke physical activity: A mixed-methods feasibility study Supplemental material, sj-docx-11-cre-10.1177_02692155251382502 for Tailored implementation of a behaviour change intervention for post-stroke physical activity: A mixed-methods feasibility study by Sarah A Moore, Jessica Calder and Sebastian Potthoff in Clinical Rehabilitation
