# Supplementary material for: Tailored implementation of a behaviour change intervention for post-stroke physical activity: A mixed-methods feasibility study
Source: Clin Rehabil. 2025 Oct 3;39(12):1589–605. doi: 10.1177/02692155251382502 (PMC12615851; doi:10.1177/02692155251382502)
Supplement: sj-pdf-1-cre-10.1177_02692155251382502 - Supplemental material for Tailored implementation of a behaviour change intervention for post-stroke physical activity: A mixed-methods feasibility study [file sj-pdf-1-cre-10.1177_02692155251382502.pdf]

Physical Activity Routines
After Stroke

Northumbria Healthcare
NHS Foundation Trust

Components of Physical Activity Routines After Stroke (PARAS) programme

Academic Health Science Network
North East and North Cumbria

Northumbria University
NEWCASTLE

Newcastle University

Components of Physical Activity Routines After Stroke (PARAS) programme

TIDieR component	Description
**What: materials**	• Workbook<br>• Self-monitoring tools (physical activity diaries, outcome measures, commercially available apps)<br>• Goal setting summary sheet<br>• Healthcare professional repository of local physical activity options
**What: procedures**	Supported self-management programme including the following behaviour change techniques (BCTs): information about health consequences; salience of consequences; social support (unspecified); instruction on how to perform the behaviour; demonstration of the behaviour; goal setting (behaviour); problem solving; information about antecedents; monitoring of emotional consequences; information about health consequences; information about social and environmental consequences; social reward; habit formation; credible source; action planning; feedback on behaviour and self-monitoring of behaviour.
**Who**	**Who is PARAS aimed at?**<br>• Adult stroke survivors who have agreed with a healthcare professional that there is potential for them to increase their daily physical activity and/or reduce their daily sitting time<br>• The stroke survivor should be physically and cognitively able to safely self-manage physical activity (this could be with a carer) and should have no contraindications for undertaking physical activity including being told by their GP or consultant that they should not take part in physical activity<br>**Who should deliver PARAS?**<br>A healthcare professional or other professional who is a credible source (e.g. well informed on stroke rehabilitation) and plays a key role in the stroke survivors rehabilitation e.g. physiotherapist, occupational therapist, technical instructor, rehabilitation worker, charity worker, fitness instructor.
**How**	**Part 1:** Preparation: paperwork can be sent out to the patient, or discussed face-to-face.<br>**Part 2:** Goal setting, action planning, barrier identification, coping planning: ideally delivered face-to-face but could be delivered remotely if assessed as safe.<br>**Part 3:** Reviewing progress: delivered face-to-face or remotely.

TIDieR component	Description
**Where**	Inpatient, outpatient or home and community settings
**When and how much**	When PARAS is delivered will vary dependent on wishes/needs of the patient and health care professional's opinion on the best timing/ availability of resources.<br>The three parts must be completed but the number of sessions required by the stroke survivor will depend on individual needs. Some will be able to undertake part 1 independently and only need a single session for part 2 and 3. Others may need more sessions particularly if original goals are not achieved, or there is a need for new goals. There is no upper limit to sessions, the number is defined by patients' support needs/availability of resources.
**Tailoring to participants**	PARAS support is graded to individual ability, preference, and values and progressed as able.
**How well**	Healthcare professionals delivering the PARAS programme should only be able to access the PARAS toolkit if they have completed the training videos. The skills of the healthcare professional will be reviewed after delivering the programme with patients to aid delivery.

The components of the PARAS programme were systematically designed and tested. This process included the following steps;

1	Qualitative study with stroke survivors, informal carers and healthcare professionals to identify barriers and enablers to physical activity after stroke
2	Systematic review identifying promising components of the PARAS intervention from previous studies
3	Stroke survivor and informal carer co-design workshops to test prototype PARAS intervention
4	Feasibility, acceptability and fidelity study testing PARAS delivery in three community stroke services
5	Stroke survivor and healthcare professional feedback on iterative changes to PARAS programme in response to the feasibility study
