# Supplementary material for: Tailored implementation of a behaviour change intervention for post-stroke physical activity: A mixed-methods feasibility study
Source: Clin Rehabil. 2025 Oct 3;39(12):1589–605. doi: 10.1177/02692155251382502 (PMC12615851; doi:10.1177/02692155251382502)
Supplement: sj-docx-2-cre-10.1177_02692155251382502 - Supplemental material for Tailored implementation of a behaviour change intervention for post-stroke physical activity: A mixed-methods feasibility study [file sj-docx-2-cre-10.1177_02692155251382502.docx]

**Appendix B Focus group script**

**Introduction**

- Confirmation of consent and confidentiality of the information.
- Gather demographic data
- General introductions and icebreaker activities if required.
- Introduce the purpose of the interview/focus group.
- Brief presentation providing an overview of PARAS.

**Knowledge and interest in PARAS**

1. *How many people had heard about PARAS before this current research?*
2. *For those that have heard about PARAS how and when did you first hear about it?*
3. *What made you interested in PARAS?*

*Prompt: explore value of PARAS, what prompted interest*

1. *What might make others interested in using PARAS in the future?*

**Initiating use of PARAS**

1. For those of you who have heard about it have any of you had a go at using PARAS or parts of PARAS?
2. What helped you start using it?
3. If you haven’t used PARAS what might help you to start?

Prompt: Training, resource gathering, champion

1. What might stop you having a go at using PARAS?

**Use of PARAS**

1. *If you are currently using PARAS how are you using it?*
2. *If you are not using PARAS how do you think it could be used?*

*Prompt: explore how PARAS is typically used and how often, methods of monitoring success, PARAS adaptations, other forms of physical activity / goal setting support.*

1. *Have you received any feedback from patients about PARAS and your delivery of PARAS?*

**Embedding PARAS into daily practice**

1. *Would you say PARAS is part of your daily practice?*

*If yes 12a.* ***if part of daily practice*** *how long did this take?*

*Do you have a clear plan for when, where and how you will use PARAS. Is there anything that prompts you to use it (e.g. particular patient characteristics). Is there anything that gets in the way of you using it?*

*Do you have any plans to enable the continued use of PARAS?*

***If no 21b.if not part of daily practice*** *why do you think this might be?*

1. *If you haven’t used PARAS what do you think would enable it to become part of daily practice from an individual perspective?*

*Prompt resources, plans, goals, feedback, evaluation, audit*

*13b. and from a team perspective?*

1. *Do you think it would be helpful to adapt or create any new PARAS resources / training to aid implementation?*
2. *Are you considering using PARAS as a team in the future? Would you be interested in working together to create a plan for implementation as part of the next phase of the project?*
3. *Would you be interested in taking some workbooks to have a go with PARAS?*
