# Supplementary material for: Tailored implementation of a behaviour change intervention for post-stroke physical activity: A mixed-methods feasibility study
Source: Clin Rehabil. 2025 Oct 3;39(12):1589–605. doi: 10.1177/02692155251382502 (PMC12615851; doi:10.1177/02692155251382502)
Supplement: sj-docx-3-cre-10.1177_02692155251382502 - Supplemental material for Tailored implementation of a behaviour change intervention for post-stroke physical activity: A mixed-methods feasibility study [file sj-docx-3-cre-10.1177_02692155251382502.docx]

**Appendix C**

**Implementation plan V1**

Overall goal:

| Areas of focus / objectives | Implementation strategies selected | Actions required to operationalise strategies (consider, who, what, when/how, where) | Measure of outcome  (consider method, how often, how this will be disseminated) | Potential barriers and coping plans |
| --- | --- | --- | --- | --- |
|  |  |  |  | If:  Then: |
|  |  |  |  | If:  Then: |
|  |  |  |  | If:  Then: |
