# Supplementary material for: Tailored implementation of a behaviour change intervention for post-stroke physical activity: A mixed-methods feasibility study
Source: Clin Rehabil. 2025 Oct 3;39(12):1589–605. doi: 10.1177/02692155251382502 (PMC12615851; doi:10.1177/02692155251382502)
Supplement: sj-docx-4-cre-10.1177_02692155251382502 - Supplemental material for Tailored implementation of a behaviour change intervention for post-stroke physical activity: A mixed-methods feasibility study [file sj-docx-4-cre-10.1177_02692155251382502.docx]

**Appendix C Implementation plan V2**

**Name / team: Date:**

**Action plan to achieve your goal using the strategies:** Consider *what, how, who, when e.g. tailor PARAS for ward, who will deliver, to whom, how to record?*

**Implementation strategies (using taxonomy) to support short term goal:** *e.g. promote adaptability; change physical structure and equipment; identify and prepare champions*

**Potential barriers:** *e.g. If-then plans*

**Outcome:** *How will you determine if your implementation plan has been successful? E.g. number of therapists/ patients using PARAS, patient testimonials, outcome measures*

**Short-term PARAS implementation SMART goal:** *e.g. To tailor PARAS resources to enable use with stroke inpatients alongside current MDT led goal setting 3/12.*
