# Supplementary material for: Tailored implementation of a behaviour change intervention for post-stroke physical activity: A mixed-methods feasibility study
Source: Clin Rehabil. 2025 Oct 3;39(12):1589–605. doi: 10.1177/02692155251382502 (PMC12615851; doi:10.1177/02692155251382502)
Supplement: sj-docx-5-cre-10.1177_02692155251382502 - Supplemental material for Tailored implementation of a behaviour change intervention for post-stroke physical activity: A mixed-methods feasibility study [file sj-docx-5-cre-10.1177_02692155251382502.docx]

**Appendix D**

**PARAS – List of implementation strategies**

This list includes a comprehensive list of discrete implementation strategies that could be applied to enable the implementation of PARAS within your stroke service. For each identified goal this list can be used to highlight strategies to enable achievement of this goal.

| **PLAN STRATEGIES** |  |
| --- | --- |
| *STRATEGY* | *DEFINING CHARACTERISTICS* |
| BUILD BUY-IN |  |
| Identify and prepare champions | Identify and prepare individuals within the organisation who dedicate themselves to supporting, marketing, and driving through an implementation, overcoming indifference or resistance that the intervention (i.e. PARAS) may provoke in an organization |
| Involve executive boards | Involve existing governing structures (e.g., clinical leadership, policy makers) in the implementation effort, including the review of data on implementation processes |
| Involve patients/consumers and family members | Engage or include patients/consumers and families in the implementation effort |
| DEVELOP RELATIONSHIPS |  |
| Build a coalition | Recruit and cultivate relationships with partners in the effort to implement PARAS e.g building community of practice |
| **EDUCATE STRATEGIES** |  |
| *STRATEGY* | *DEFINING CHARACTERISTICS* |
| DEVELOP MATERIALS |  |
| Develop educational materials | Develop and format manuals, toolkits, and other supporting materials in ways that make it easier for stakeholders to learn about PARAS and for clinicians to learn how to deliver PARAS |
| EDUCATE |  |
| Conduct educational meetings | Hold meetings targeted toward different stakeholder groups (e.g., broader MDT, funders, patient/consumer, and family stakeholders) to teach them about PARAS |
| Conduct ongoing training | Plan for and conduct training in PARAS in an ongoing way e.g. booster training, testing competence |
| Distribute educational materials | Distribute educational materials (including guidelines, manuals, and toolkits) in person, by mail, and/or electronically |
| Use train-the-trainer strategies | Train designated clinicians or organizations to train others in PARAS |
| EDUCATE THROUGH PEERS |  |
| Create a learning collaborative | Facilitate the formation of groups of providers or provider organizations and foster a collaborative learning environment to improve implementation of PARAS e.g. online community |
| Identify early adopters | Identify early adopters at the local site to learn from their experiences with PARAS |
| Inform local opinion leaders | Inform providers identified by colleagues as opinion leaders or “educationally influential” about PARAS in the hopes that they will influence colleagues to adopt it |
| Promote network weaving | Identify and build on existing high-quality working relationships and networks within and outside the organization, organizational units, teams, etc. to promote information sharing, collaborative problem-solving, and a shared vision/goal related to implementing PARAS |
| INFORM & INFLUENCE STAKEHOLDERS |  |
| Increase demand | One way of increasing demand is to educate patients about PARAS so that they demand it from their providers (e.g. advertise via posters) |
| Prepare patients/consumers to be active participants | Prepare patients/consumers to be active in their care, to ask questions, and specifically to inquire about care guidelines, the evidence behind clinical decisions, or about available evidence-supported treatments |
| **FINANCE STRATEGIES** |  |
| *STRATEGY* | *DEFINING CHARACTERISTICS* |
| Access new funding | Access new or existing money to facilitate the implementation of PARAS e.g. funding for workbooks |
| **RESTRUCTURE STRATEGIES** |  |
| *STRATEGY* | *DEFINING CHARACTERISTICS* |
| Change physical structure and equipment | Evaluate current configurations and adapt, as needed, the physical structure and/or equipment (e.g., ensuring there is saved copy of the workbook on a shared drive and printed copies that can be accessed when in community) of workbook) to best accommodate the delivery of PARAS |
| Change record systems | Change records systems to allow better assessment of implementation or clinical outcomes e.g. audit use, change current eRecords to include ability to record use of PARAS |
| Promote adaptability | Identify the ways PARAS can be tailored to meet local needs and clarify which elements of PARAS must be maintained to preserve fidelity e.g. use sections relevant to inpatient care |
| **QUALITY MANAGEMENT STRATEGIES** |  |
| *STRATEGY* | *DEFINING CHARACTERISTICS* |
| Audit and provide feedback | Collect and summarize clinical performance data in relation to PARAS over a specified time period and give it to clinicians and administrators to monitor, evaluate, and modify provider behavior e.g. audit use of PARAS and collection of outcomes |
| Capture and share local knowledge | Capture local knowledge from implementation sites on how implementers and clinicians made PARAS work in their setting and then share it with other sites e.g. sharing success stories or how PARAS was tailored to a specific area |
| Develop and implement tools for quality monitoring | Develop, test, and introduce into quality-monitoring systems the right input—the appropriate language, protocols, algorithms, standards, and measures (e.g. using peer review of delivery of PARAS ensuring all key components covered use of OARS skills) |
| Obtain and use patients/consumers and family feedback | Develop strategies to increase patient/consumer and family feedback on PARAS (e.g. collect patient testimonials or feedback on PARAS) |
| Organize clinician implementation team meetings | Develop and support teams of clinicians who are implementing PARAS and give them protected time to reflect on the implementation effort, share lessons learned, and support one another’s learning |
| Provide clinical supervision | Provide clinicians with ongoing supervision focusing on PARAS (e.g. supervision joint sessions with junior staff). Provide training for clinical supervisors who will supervise clinicians who provide PARAS |
| Remind clinicians | Develop reminder systems designed to help clinicians to recall information and/or prompt them to use PARAS (e.g. email reminders) |
