# Supplementary material for: Tailored implementation of a behaviour change intervention for post-stroke physical activity: A mixed-methods feasibility study
Source: Clin Rehabil. 2025 Oct 3;39(12):1589–605. doi: 10.1177/02692155251382502 (PMC12615851; doi:10.1177/02692155251382502)
Supplement: sj-docx-7-cre-10.1177_02692155251382502 - Supplemental material for Tailored implementation of a behaviour change intervention for post-stroke physical activity: A mixed-methods feasibility study [file sj-docx-7-cre-10.1177_02692155251382502.docx]

**Appendix E Example Implementation Plan 2a**

**Name Date: 5^th^ September 2024**

**Potential barriers:** *e.g. If-then plans*

*Lack of time : book out specific time in diary and discuss with colleagues a plan
Lack of confidence / understanding of the principles : discuss with champions / peers*

**Outcome:** *How will you determine if your implementation plan has been successful? E.g. number of therapists/ patients using PARAS, patient testimonials, outcome measures*

100% of OTs, PTs and RAs within CNRS will have used PARAS with at least one patient and completed a reflective form
Self reported improved confidence in implementing PARAS from individuals baseline rating and rating after implementation plan 1.

**Action plan to achieve your goal using the strategies:** Consider *what, how, who, when e.g. tailor PARAS for ward, who will deliver, to whom, how to record?*

*Circulate booklets –(name)
Complete implementation plan 1
Identify appropriate patients
provide reflection template*

*monitor progress within individual clinical supervision – all*

**Implementation strategies (using taxonomy) to support short term goal:**

*Identify and Prepare champions – [therapist name] (North) and [Therapist name] (South team)*

*Conduct ongoing training*

*Create a learning collaborative*

*Identify early adopters – (name)*

*Provide clinical supervision*

*Remind clinicians
change physical structure and equipment
promote adaptability*

**Short-term PARAS implementation SMART goal:**

For everyone within [Team name] OT and PT to have trialed PARAS on 1 or 2 patients using the materials provided in the booklet or on the website by the end of end of November 2024.
