# Supplementary material for: Tailored implementation of a behaviour change intervention for post-stroke physical activity: A mixed-methods feasibility study
Source: Clin Rehabil. 2025 Oct 3;39(12):1589–605. doi: 10.1177/02692155251382502 (PMC12615851; doi:10.1177/02692155251382502)
Supplement: sj-docx-8-cre-10.1177_02692155251382502 - Supplemental material for Tailored implementation of a behaviour change intervention for post-stroke physical activity: A mixed-methods feasibility study [file sj-docx-8-cre-10.1177_02692155251382502.docx]

**Appendix D Example Implementation plan 2b**

**Implementation plan**

**Name / team: [Team name] Date: 5^th^ September 2024**

**Implementation strategies (using taxonomy) to support short term goal:**

*Identify and Prepare champions – [Therapist Name] (North) and [Therapist Name} (South team)*

*Conduct ongoing training*

*Distribute educational materials*

*Create a learning collaborative*

*Identify early adopters – [Therapist Name] and [Therapist Name]*

*Provide clinical supervision*

*Remind clinicians*

**Action plan to achieve your goal using the strategies:** Consider *what, how, who, when e.g. tailor PARAS for ward, who will deliver, to whom, how to record?*

*Allocate protected time - all
circulate booklets –XXXXX
monitor progress within individual clinical supervision – all*

**Potential barriers:** *e.g. If-then plans*

*Lack of time : book out specific time in diary and discuss with colleagues a plan
Lack of understanding the material : discuss with colleagues more familiar with PARAS / PARAS champions*

**Outcome:** *How will you determine if your implementation plan has been successful? E.g. number of therapists/ patients using PARAS, patient testimonials, outcome measures*

100% of OTs, PTs and RAs within [Team name]will have completed the online training
100% of OTs, PTs and RAs within [Team name] will be familiar with the content of the PARAS booklet
Self reported improved confidence in implementing PARAS from individuals baseline rating

**Short-term PARAS implementation SMART goal:**

For everyone within [Team name] OT and PT to have completed the 2 hrs of training videos on the PARAS website by the 18^th^ October 2024.
