# Supplementary material for: Tailored implementation of a behaviour change intervention for post-stroke physical activity: A mixed-methods feasibility study
Source: Clin Rehabil. 2025 Oct 3;39(12):1589–605. doi: 10.1177/02692155251382502 (PMC12615851; doi:10.1177/02692155251382502)
Supplement: sj-docx-9-cre-10.1177_02692155251382502 - Supplemental material for Tailored implementation of a behaviour change intervention for post-stroke physical activity: A mixed-methods feasibility study [file sj-docx-9-cre-10.1177_02692155251382502.docx]

**Appendix F**

Categories, sub-categories and exemplar quotes relating to barriers and facilitators to implementation plans

| Categories | Sub-categories | Exemplar quotes |
| --- | --- | --- |
| Motivation to implement PARAS | When PARAS aligns with team priorities / philosophy / skills implementation is more likely | [Facilitator to Team 4 implementation plan] *‘…it being clinically relevant to the people I was using it with.’* (Band 7, PT, review)  [Facilitator to team 4 implementation plan] *‘…Our service has a philosophy of behaviour change and have been trying to use this as our model for over 5 years now’.* (Band 7, PT team characteristics)  [Facilitator to team 4 implementation plan] *‘…I think a lot of the content and the delivery was quite familiar. So we were quite comfortable with a lot of the language that was used around sort of barriers, facilitators, goals’* (Band 7, PT, review)  [Barrier to Team 3 implementation plan] *‘…kind of priorities as well, like team priorities’* (Band 7, PT, review)  [Barrier to Team 1 implementation plan] *‘…We just haven't had the capacity to look at this since our last meeting. It's on my list of things to do once things quieten down a bit.’* (Band 7, OT, email)  [Facilitator to Team 4 implementation plan] *‘…I do think that having that variety of it wasn't just exercise, it was very much activity… it made them think about it in a different way.* (Band 6, PT, review) |
|  | Sharing PARAS success stories aids motivation to implement PARAS | *‘Our team definitely like good patient feedback…..That is an absolute driver in our team.’* (Band 7, PT, workshop)  *‘…we can ask them how they like. We could almost ask the patient at the end like how they found it. Have they found it beneficial… and then feed that back to the team. (Band 7, PT, workshop)*  [Facilitator Team 1 after hearing a successful patient PARAS story] *‘…Yeah, and actually, because I've been thinking of someone one’. (Band 7 OT workshop)*  [Barrier to Team 3 implementation plan] ‘…I think certainly something from this that haven't really done is kind of getting that feedback… if you know if someone was to do the training and then utilize that, getting the feedback from the patient, but also the feedback from the therapist on how they found that.’ (Band 7, PT, review)  [Facilitator to Team 4 implementation plan] *‘…I think the pictures were brilliant because you could just show them the pictures. You didn't have to speak about it.’* (Band 7, PT, review)) |
| Stakeholder involvement in PARAS implementation | Core team engagement enables PARAS implementation | [Barrier to Team 2 implementation plan] *‘…at the minute it is just completely reliant upon certain individuals being around and then being free enough to kind of take the time to deliver.’* (Band 8a, PT, review)  [Barrier to Team 2 implementation plan] *‘…I know it would be a massive extra bit of work, but I don't know whether or not kind of get [Team name who were not involved at start of implementation process] involved.’* {Band 7, PT, review)  *‘If it’s a team effort then it feels more manageable.’* (Band 7, PT, workshop)  *‘Chance to discuss how it would work for our individual team.’* (questionnaire response) |
|  | Multi-professional team awareness of PARAS aids implementation | [Barrier to Team 3 implementation plan] *‘…knowing about it would have helped. And actually I think it probably would because even at this point, if I was to bring it up at an MDT I’d probably get a lot of blank faces’. (Band 6, PT, review)*  *‘…I think it’s about educating everybody though, isn’t it? It’s about introducing it to not just the physios but obviously the OTs as well and even the speech and language therapists’ (Band 7, PT, workshop)*  *‘…It's kind of having some of those discussions in MDT and getting our TI’s possibly involved in identifying activities.’ (Band 7, OT, workshop)* |
| Leadership and planning of PARAS implementation | Implementation facilitators support implementation | *‘…It being facilitated by [the research team]’* (questionnaire response)  *‘Little bit of a nudge how you're getting on [from the facilitator] probably does help.’ (Band 7 OT, workshop)*  *‘You [the facilitator] sent that e-mail because of that I took note’ (Band 7 OT, workshop)*  *‘In our brains and yeah, we're thinking about it’ (Band 6 PT workshop)*  *‘…Yeah, even once a month, just to what's not to go. Yeah, just checking in. How's your target going? Yeah. Monthly reminder. Polite reminder. Because I do think it'll go off on radar.’* (Band 7, PT, workshop) |
|  | Identifying PARAS champions enables implementation success | *[facilitator to Team 4 implementation plan] ‘It was really helpful to have a champion…* *So within the team to somebody I knew that I that was kind of checking in, giving the prompts I could go to, I could ask the questions. They were knowledgeable.’* (Band 6 PT, review)  *‘…Having [X] and [X] as champions to prompt, remind and encourage us through the implementation process.’* (Band 6, PT, questionnaire)  [Barrier to Team 3 implementation plan] *‘…I think ultimately I genuinely haven't kind of pushed this forward probably strongly enough…. I haven't. Kind of enforced that with my staff, so I definitely feel that I haven't led on that very well.’* (Band 7, PT, review)  [Barrier to Team 2 implementation plan] *‘…Sounds like it was useful. Having X as kind of a identified person to run with it at X, …Here we haven't really ever identified anyone and had that sort of main person’* (Band 7 PT review) |
|  | Forming clear tailored implementation plans aids implementation | *‘Good to logically work through a plan’* (questionnaire response)  *‘I think. It works [focus group followed by workshop]’* (Band 7 PT, workshop)  [Barrier to Team 3 implementation] *‘…I think firstly, I should have got this written up immediately and had it as more of a priority…. I wonder if you know a plan was there earlier. Whether that might have changed things.’* (Band 7, PT, review)  [Barrier to Team 2 implementation plan] *‘…I think we just need to build that in there and maybe have it auditable so that it's rather than just being recorded somewhere and falling on lots of individuals, we have a clear process about how we're going to use it,* (Band 8a, PT, review) |
|  | Implementation plan reminders / prompts enable implementation | [Facilitator to Team 4 implementation plan] ‘*…just a weekly reminder of who's starting it, with who and how its going.’* (Band 6 PT, review)  [Facilitator to Team 4 implementation plan] *‘…I did send some reminders around via e-mail. Because I think when you're very busy with lots of other things, it can slip off the radar’* (Band 7, PT, review)  [Barrier to Team 3 implementation plan] *‘…we do have a bit of like an MDT sheet that we go through and we're looking at kind of outcome measures. And I just wonder if that could be kind of added on as a consideration.’* (Band 7, PT, review) |
|  | Having suitable PARAS resources available enables implementation | *‘I think we definitely don't carry the resources or. Have the resources we don't.’* (Band 6, PT, workshop)  *‘…so probably carrying resources well, if we're there in the conversation arises, we can have that conversation there and then yeah, having that toolkit.’* (Band 7 PT, workshop)  [Facilitator to Team 4 implementation plan] *‘…We've got some booklets that were in like, each of the places, so some of those, I'm sure there's more we could do to make it practically easier to use it, but that's sort of where we started.* (Band 7, PT review)  [Facilitator to Team 4 implementation plan] *‘…she went on and saved the documents into a folder, made them editable for us so that we could email’* (Band 7, PT, review) |
| PARAS delivery skill acquisition | Training and use of PARAS can enable implementation and fidelity | [Barrier to Team 2 implementation plan] *‘…Getting that member of staff who's keen to do it, but they're making sure that they're in a position where they're comfortable working with, instruct to be able to do this and they're making sort of good decisions around which patients are using it and which ones aren't.’* (Band 8a, PT, review)  [Facilitator to Team 4 implementation plan] *‘…we just went through one or two sections [of the PARAS training website] per week and felt that actually then also because we were together as a team it just allowed us to quickly kind of agree the plan.’* (Band 6, PT, review)  [Facilitator to Team 4 implementation plan] *‘…I think what it showed is that you could do the training differently depending on what fitted best with your structure, and both seem to go quite well.’* (Band 6, PT, review)  [Facilitator to Team 2 implementation plan] *‘I think it's probably… easier for me because I was involved in the pilot. So I feel I'm quite used to it from kind of doing it from early on. So I think probably that makes maybe does make it a little bit easier for me to do it.’* (Band 6 PT, review) |
|  | Training and making rotational staff champions can aid implementation | [Facilitator to Team 3 implementation] *‘…We have tasked our current B5s [junior staff] with this project so you will hopefully see a few names sign up for the PARAS Training.*’ (Band 7, PT, email)  [Facilitator to Team 2 implementation plan] *‘…It did work quite well when [rotational member of staff] was able to pick them and I think we did it so that I did the initial one with him. And then and he did do another one by himself.’* (Band 6, PT, review)  *‘Coming on kind of as a new member of staff. Like, I mean, you probably haven't seen the website, but it might be worth just having a look at the website and like maybe having just sign posting new members of staff to it’* (Band 6 PT workshop)  *‘…So maybe we do need a little bit of that to make sure that kind of the rotational stuff are up to date, because I suppose there's a bit about the fidelity of the delivery’* (Band 7 OT workshop)  *‘To do the bit training and. And then setting them and then they just. Have to start small so they start with one.’ (Band 6, PT, focus group)* |
|  | Becoming familiar with PARAS aids self-efficacy and ability to adapt delivery | *‘I think that's why I chose these certain people as well because. I wasn't familiar with it, so that was a good opportunity to kind of try it’ .* (Band 6 PT workshop)  [Facilitator to Team 4 implementation plan] *‘…we all have quite a quite a group of stroke clients on our caseload, but for some reason we've chose to not use it with everyone…within our service tend to group clients into readiness for rehab. So for those green clients, actually, we've not really been using it because they're already doing activity’ so it was more those amber that we've utilised it with.’* (Band 6, PT, review)  [Facilitator to Team 4 implementation plan] *‘…I think it having that ability to vary it means that you can use elements of it even if maybe not the full program, certainly with [problems with] communication and cognition . …We had those conversations as a session rather than like giving [the booklet], whereas another gentleman I tried it with cognitively great. He he took the book away and he did it’* (Band 7, PT, review)  [Barrier to Team 1 implementation plan] *‘I think if I had been a little bit more familiar with the programme. Probably I could have adapted it, but at the minute I'm not’. (*Band 7 OT workshop) |
|  | Peer support and clinical supervision aids PARAS implementation | [Facilitator to Team 4 implementation plan] *‘…a lot of it seems to have developed over these sharing of a patient or discussing it after a patient or just reflecting in conversation, doing the training with someone else or alongside your team maybe.’* (Band 7 PT review)  [Facilitator to Team 4 implementation plan] *‘…it was really helpful because [X] and I shared a patient and actually we could then chat about how it was going in terms of introducing it with him and sort of how well he was getting on and sort of...So it's quite nice to be able to sort of two heads together.’* (Band 7 PT review)  [Facilitator to Team 2 implementation plan] *‘…It did work quite well when [X] was able to pick them and I think we did the initial one with him*. (Band 6, PT, review)  *‘might be quite good just to share experiences’ (Band 7 OT workshop)*  [Solution to barrier to Team 3 implementation plan]*‘…I think it would be really helpful to share [other people’s implementation plans]* (Band 7, PT, review) |
