# Supplementary material for: Tailored implementation of a behaviour change intervention for post-stroke physical activity: A mixed-methods feasibility study
Source: Clin Rehabil. 2025 Oct 3;39(12):1589–605. doi: 10.1177/02692155251382502 (PMC12615851; doi:10.1177/02692155251382502)
Supplement: sj-docx-10-cre-10.1177_02692155251382502 - Supplemental material for Tailored implementation of a behaviour change intervention for post-stroke physical activity: A mixed-methods feasibility study [file sj-docx-10-cre-10.1177_02692155251382502.docx]

**Appendix G –Framework and rationale for logic model for future PARAS tailored implementation approach**

The logic model for future delivery of the tailored implementation approach for PARAS is built upon the initial approach used in this study (Figure 1) which has been iteratively developed based on study findings.

**Figure 1** Tailored implementation process applied in study


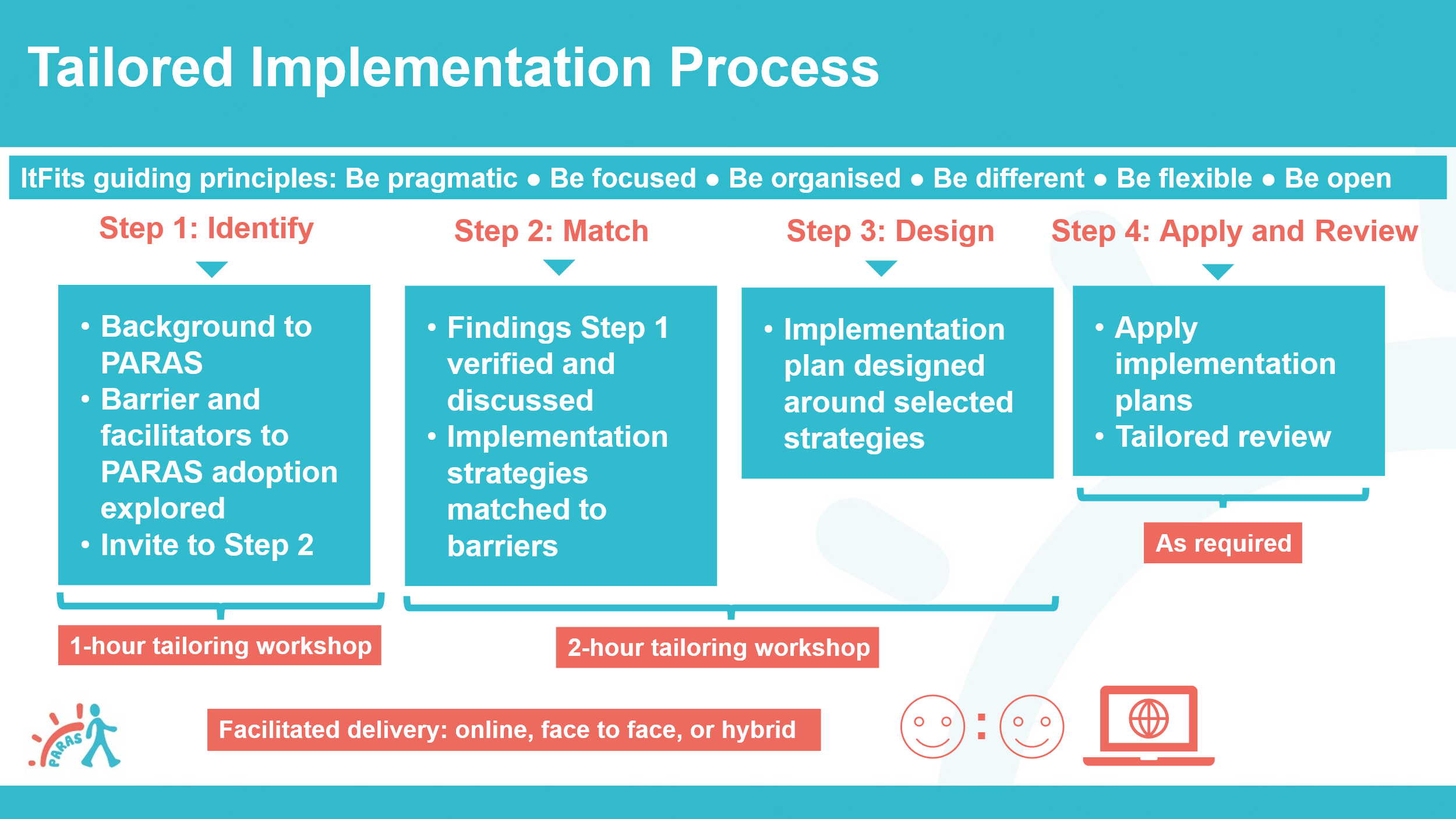


**Rationale and components of logic model**

The components of the logic model (Figure 2) and their rationale will now be described.

**Figure 2** Logic model for future PARAS tailored implementation approach


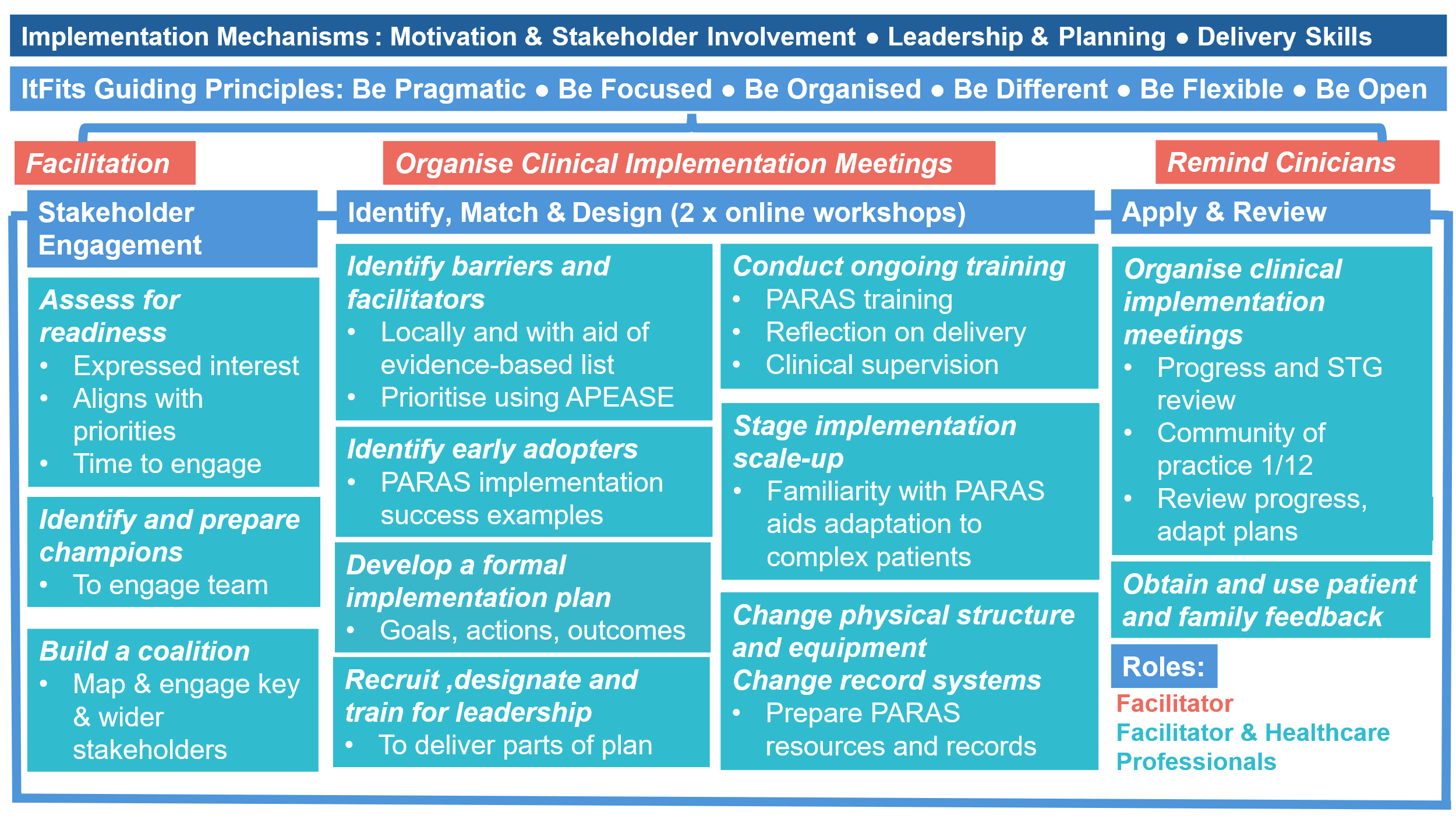


1. **Key implementation mechanisms (depicted in dark blue in the model)**

The key implementation mechanisms identified in the study that were linked with implementation plan success included: motivation and stakeholder involvement, leadership and planning and delivery skill acquisition. These mechanisms overarch the model and have been used to map implementation strategies selected.

1. **ItFits framework and guiding principles (depicted in sky blue in the model)**

Our study demonstrated it was feasible to apply the ItFits toolkit as a framework for our approach embedding the four stages: 1. Identify; 2. Match; 3. Design; and 4. Apply and Review (Table 1) and the six ItFits guiding principles: 1) Be pragmatic; 2) Be focused; 3) Be; 4) Be different; 5) Be flexible and 6) Be open. As this approach was found to be feasible it has been embedded within the logic model with operationalisation informed by our findings. Alongside the four stages we have emphasised the importance of stakeholder engagement particularly at the beginning, but also throughout the four stages. We did not initially include stakeholder engagement within our framework of delivery of the tailored implementation. However, one of the key barriers to implementation plan success in our study was when core members of the team were not involved or when wider members of the multi-disciplinary team were not aware of implementation plans.

In contrast to the ItFits toolkit what we did not include within our approach was specific guidance on reviewing plans and a time frame for delivery, instead leaving this open for teams to decide. In retrospect this may have been a barrier to success of implementation plans and a more specific mechanism of review has been embedded within the model. Teams will be asked to deliver plans across 6 months, monitoring and adopting plans if required. Implementation plan delivery decisions will be informed with the options provided in the ItFits toolkit: Stop: conclude the work on implementation if it has been successful; Continue: keep going with your current strategy (go to apply and review - monitor the delivery of your plan); Change strategy: shift your focus to work on a different strategy to help overcome your current goal and barrier (go to match); Change barrier focus: switch your focus to address a different barrier (go to identify); Change goal focus: change your focus to work on a different goal (go to identify).

With regards to the guiding principles the main iterative developments that have been made in response to the study findings are the provision of tools to enable embedding of principles. Within the study, teams that created SMART goals with clear action plans had more success. To enable this process and the principles of being pragmatic and focused teams will be encouraged to use the APEASE criteria (Acceptability, Practicability, Effectiveness, Affordability, Side-effects/Safety, and Equity) when formulating plans.(Michie, Atkins, & West, 2014) Some teams potentially tried to target too many barriers to adopting PARAS in the study which may have influenced success. In the future it is important for teams to create a hierarchy of barriers they are confident they can address to inform focus. We had to create a new version of the implementation plan during the study as the first team struggled to formulate clear plans with the first version. Although one team used the template very well in the study to ‘be organised’ the other two teams could potentially have produced clearer plans if the paperwork had better supported the process. A third version has been created to enable this process.

As this was the first exploration of implementation of PARAS in real world settings, we had no examples of determinants of practice and potential matching implementation strategies to provide to teams to enable them to ‘be different’. Having these examples was highlighted by teams as a way of enabling development of plans. This current study has enabled the development of a repository of PARAS specific evidence-based determinants of practice which will be used by teams to aid development of plans. Being flexible was an important part of design and delivery of implementation plans. Although teams were based in the same NHS trust, they were often spread across different parts of the service, and it became apparent that plans needed to be tailored to address specific parts of the pathway. Some teams lost members of staff during the period of implementation and did not have coping plans to target this common problem. More emphasis will now be placed upon the creation of coping plans and alteration of plans if they are not achievable. In the study the facilitator tried to enable time teams to ‘be open’. this was difficult though when not all members of the team were engaged from the start. Mapping stakeholders and using the online delivery and tools such as break out rooms it is hoped more members of the team can be engaged and a safe space for open and honest conversations will be created to make every member of the team feel valued.

| **ItFits stages** | |
| --- | --- |
| **Stakeholder engagement** | 1) Identifying and prioritising implementation goals and barriers to reaching those goals |
|  | 2) Matching up implementation barriers to strategies |
|  | 3) Designing a plan for carrying out strategies in a local context |
|  | 4) Applying strategies and reviewing progress. Options for review: stop, continue, change strategy / goal / focus |

| **ItFits overarching principles** | PARAS operationalisation |
| --- | --- |
| 1. Be pragmatic: Focus on realistic achievable steps | The facilitator supports teams to outline clear goals and action plans. APEASE criteria guide process. |
| 1. Be focused: Focus on one thing at a time don’t try to do everything at once | Teams create a hierarchy of barriers they are confident they can address to inform where to focus. |
| 1. Be organised: each step needs an identified owner to take responsibility for delivery | Clear identification of SMART goal, action plan with assigned individuals and coping plans |
| 1. Be different: do not focus on things you feel comfortable with or the things that you would normally do | Use of evidence-based list of barriers and strategies to implementation of PARAS to enable teams to think through barriers and potential strategies and think differently |
| 1. Be flexible: the same solution may not work for everyone so be prepared to adapt your plans and ideas | Creation of comprehensive coping plans, regular review of plans applying options for review. |
| 1. Be open: listen to and value your stakeholders’ knowledge and experience | Engage correct stakeholders and create a platform where all views can be acknowledged |

1. **Mode of delivery**

In contrast to the ItFits toolkit, which is self-guided, our tailored implementation was facilitated by the research team. The teams in our study indicated it was helpful to have a facilitator to aid organisation of meetings, guide the workshops, help problem solve and to check in to review progress. Having assistance to create time and space away from clinical pressures was an important aspect of implementation plan success. The teams also appreciated having reminders from the facilitator about implementation plans. A facilitated approach will be used within the model.

The identify match and design stages of the ItFits toolkit were delivered across two workshops with the apply and review stage tailored to each teams’ needs in the current study. Attendance at face-to-face workshops proved difficult for some team members limiting engagement. Engagement was more successful when the workshops were delivered online, and the facilitator was able to use online break-out groups to aid small group discussion. As stakeholder engagement was an important implementation mechanism the future mode of delivery will be online. The initial workshop took one hour to deliver with the second taking two hours with a refreshment break these timings were feasible and will be applied to future delivery. Providing teams with the option to tailor review support did not appear to work well therefore a more structured approach will be used in future with follow-ups organised by the facilitator timed with short term goals. Participants requested in future it would be useful to get support from other teams. Monthly community of practice sessions will be organised to enable this support with key stakeholders from each service.

1. **Implementation strategies**
   1. **Implementation strategies delivered by the facilitator (depicted in orange in model)**

The following implementation strategies are applied in the logic model by the facilitator across the model

| **Implementation strategy** | **Definition according to ERIC taxonomy** | **PARAS operationalisation** |
| --- | --- | --- |
| **Facilitation** | *A process of interactive problem solving and support that occurs in a context of a recognised need for improvement and a supportive interpersonal relationship.* | The facilitator has knowledge of PARAS and supports the team through the different stages of the model through email, phone and online support. |
| **Organise clinical implementation meetings** | *Develop and support teams of clinicians who are implementing the innovation and give them protected time to reflect on the implementation effort, share lessons learned, and support one another’s learning.* | The facilitator works with team leads to organise the workshops and reviews timetabled to fit with clinical demands |
| **Remind HCP** | *Develop reminder systems designed to help clinicians to recall information and/or prompt them to use the clinical innovation.* | The facilitator contacts teams via email to remind them of any deadlines set in implementation plans |

**Implementation strategies undertaken by the facilitator and HCPs (depicted in turquoise in model)**

1. **Stakeholder engagement**

The following implementation strategies fall under early stakeholder engagement: assess for readiness, identify and prepare champions and form a coalition (see table below). In the study individuals and teams were invited to take part of they had an interest in PARAS as demonstrated by previous training or membership on the PARAS website. This helped assess for readiness to undertake PARAS implementation. Motivation to take part in the implementation process was an important determinant of success alongside alignment with priorities. When priorities were focused on clinical demands and other areas of change it reduced chances of success. A similar assessment to determine motivation to implement PARAS will be conducted for future teams and individuals. Champions were selected by teams organically within the study with some teams not identifying a formal champion impacting upon achievement of plans. The importance that was attributed to the success of implementation plans based on champions has indicated a need for a more formal process of identification and the need for more leadership roles to reduce reliance on certain individuals. Engagement from key stakeholders and wider members of the MDT is essential to enable success of plans rather than as occurred in the study where in certain teams implementation was down to a few individuals and when any of these individuals’ left plans were stalled.

| **Implementation strategy** | **Definition according to ERIC taxonomy** | **PARAS operationalisation** |
| --- | --- | --- |
| **1.1 Assess for readiness** | *Assess various aspects of an organisation to determine its degree of readiness to implement* | Individuals or teams who are interested in implementing PARAS will be invited to take part and priorities of the teams explored with the facilitator through informal discussion and a more formal assessment of whether PARAS aligns with the clinical services’ priorities and whether the service has time to engage in the process |
| **1.2 Identify and prepare champions** | *Identify and prepare individuals who dedicate themselves to supporting marketing and driving through an implementation overcoming indifference or resistance that the intervention may provoke in an organisation* | Teams will be asked to formally identify a champion to lead the process and engage in future community of practice events  Different champions will be identified for different parts of the pathway and a deputy champion nominated to cover if the champion is on leave or leaves the service. |
| **1.3 Build a coalition** | *Recruit and cultivate relationships with partners in the implementation effort* | Key and wider stakeholders will be mapped with support from the facilitator and a coalition of individuals that will aid the implementation effort formed. |

1. **Identify, match and design**

Several implementation strategies (see table below) have been embedded within the identify match and design stage which will be delivered across the first and second workshop.

As this was the first time, we had explored the implementation of PARAS in real world settings it was impossible to provide examples of barriers and facilitators to implementation. This was requested in the feedback from the study. As a result, we have compiled a list of determinants and potential matching strategies identified by teams within the first workshop of the study and from this study will provide examples of successful implementation plans which will be bult upon as more teams engage in the process.

In the current study teams identified multiple barriers and facilitators and we did not have a process of prioritisation to make implementation more manageable. Our future process will guide teams to prioritise plans using the APEASE criteria.

Some teams used the implementation plan template very well enabling success whereas others did not cover all the areas outlined in the template. A new version of the template has been created to enable teams to be more systematic (Appendix A).

One area that often teams did not outline was which individuals would undertake act as champions and who would lead on each task. As champions, leadership and planning were important determinants of success this needs to be more clearly defined.

Another area that needs prompting to consider is training. Teams that increased propositional knowledge through more formal training using the PARAS website rather than relying on experiential leaning and peer support appeared to have more success. Peer support and clinical supervision, alongside formal training enabled confidence in delivery in some teams.

Teams that naturally staged the implementation scale-up appeared to have more success as this enabled self-efficacy and motivation. For example, initially selecting patients who were potentially straight forward to develop skills before adapting delivery to more complex patients.

Ensuring any resources required for PARAS delivery were easily accessible was another important factor linked with success of plans. Teams highlighted having a structure to record use of PARAS within record systems could also enable use. These strategies have been embedded in the model.

The following implementation strategies are used in our final model within the identify, match and design stage. The facilitator works with the team to aid each step in the process:

| **Implementation strategy** | **Definition according to ERIC taxonomy** | **PARAS operationalisation** |
| --- | --- | --- |
| **2.1 Identify early adopters** | *Identify early adopters to learn from their experiences with practise innovation* | Examples of barriers and facilitators to PARAS adoption from current the study are provided to teams alongside examples of successful implementation plans |
| **2.2 Identify barriers and facilitators** | *Assess various aspects of an organisation to determine its degree of readiness to implement, barriers that may impede implementation and strengths that can be used in the implementation effort* | An introduction to PARAS is provided by the facilitator. And the teams then brainstorm potential barriers and facilitators to implementing PARAS. A list of evidence-based determinants is provided to see if anything has been missed.  Barriers are prioritised using the APEASE criteria. |
| **2.3 Develop a formal implementation blueprint** | *Develop a formal implementation blueprint that includes all goals and strategies* | Teams are supported by the facilitator to develop an implementation plan for PARAS including a short-term smart goal/s; local barriers and matched tailored implementation strategies to support the goal/s; an action plan to achieve the goal/s outlining training needs, what, when how and who will deliver each aspect of the plan and outcomes to determine implementation plan success. Any potential barriers to plans are identified alongside how these barriers could be targeted (see Appendix A) |
| **2.4 Recruit designate and train for leadership** | *Recruit designate and train leaders for the change effort* | Individuals that will lead on parts of the plan are named and any training required outlined |
| **2.5 Conduct ongoing training** | *Plan for and conduct training in the clinical innovation in an ongoing way* | A review will be made of training needs. Methods of conducting ongoing training are discussed with the team. The facilitator provides examples of how propositional knowledge, experiential learning, peer support and clinical supervision can aid implementation |
| **2.6 Stage implementation scale up** | *Phase implementation efforts by starting with small pilots or demonstration projects and gradually move to a system wide roll out* | The facilitator discusses with teams how starting with patients who are potentially more straightforward may develop confidence and skills before adapting PARAS delivery to more complex patients |
| **2.7 Change physical structure and equipment** | *evaluate current configurations an adapt as needed the physical structure and slash or equipment to best accommodate the targeted innovation* | Teams are prompted by the facilitator to think about what resources are required to aid delivery of implementation plans. For example, do teams need copies of the PARAS workbook printed out to take to patients |
| **2.8 Change record systems** | *Change record systems to allow better assessment of implementation or clinical outcomes* | Teams are prompted by the facilitator to design a method of recording use of PARAS within clinical notes |

1. **Apply and review**

During the study there was no formal review process or duration of the implementation process. Instead, teams were asked what type of tailored support they would like from the facilitator and set their own timelines. Some teams requested meetings with the facilitator aligned to short term goal timelines, whereas others requested regular e-mail prompts on use of PARAS. Feedback from the study indicated a more structured approach to the review process would be beneficial as teams that requested regular meetings with the facilitator appeared to have more success. There was also no formal system for teams to review their own progress and adapt plans accordingly. As described above under the ItFits stages implementation plan delivery decisions will be informed with the options provided in the ItFits toolkit.

Teams that met regularly to discuss progress and had a reminder system for PARAS implementation appeared more successful. Teams also indicated sharing ideas and progress across teams could be motivational. These findings have been incorporated into our model.

| **Implementation strategy** | **Definition according to ERIC taxonomy** | **PARAS operationalisation** |
| --- | --- | --- |
| **Organise clinician implementation team meetings** | *Develop and support teams of HCP who are implementing the innovation and give them protected time to reflect on the implementation effort share lessons learned and support one another's learning* | Champions and team leads organise regular implementation team meetings to discuss progress towards implementation plans. Implementation plans will be reviewed by the options provided in the ItFits toolkit e.g. Stop: conclude the work on implementation if it has been successful; Continue; Change strategy; Change barrier focus: Change goal focus.  The facilitator organises a review session/s aligned with timelines set for goals. During this review examples of success are shared and any barriers to progress discussed. If new goals are set another review is organised.  The period of implementation is 6 months.  To support collective learning the facilitator supports the development of a community of practice to share learning across teams. The community of practice is attended by team champions online once a month. |
| **Obtain and use patients/ consumers and family feedback** | *Develop strategies to increase patient/consumer and family feedback on the implementation effort* | HCP are encouraged to develop systems to capture any positive feedback from patients, friends and family on PARAS |
| **Remind clinicians** | *Develop reminder system to help clinicians to recall information and/or prompt them to use the clinical innovation* | The facilitator reminds HCPs about implementation plan timelines.  Teams develop a system to remind members to use PARAS |

**Appendix A Implementation plan template**

**Team name: Identified champions and deputies for each area:**

**PARAS implementation long term SMART goal** *e.g. All static staff trained in PARAS delivery and rotational staff training in place. PARAS considered as option and documented within clinical notes for all patients 6/12:*

**PARAS implementation short term SMART goal training (include training and delivery):** e.g**.** *All static staff completed PARAS website training and have delivered and reflected on PARAS with two patients 2/12:*

| **Local barriers***  Prioritise 3 barriers maximum | **Implementation strategies / techniques** | **How will strategy be delivered?** | **Who?** | **When by?** | **Success criteria** | **Potential barriers and coping plans** |
| --- | --- | --- | --- | --- | --- | --- |
| *e.g. Lack of knowledge and skills in PARAS delivery* | *e.g. Conduct ongoing training* | *Completion of PARAS website training* | *-All* | *1/12* | *Training completed*  *Comparison pre and post training self-rated confidence* | *If…busy with patients  Then… book time in dairy* |
| *e.g. Lack of motivation to implement PARAS amongst busy clinical schedule* | *e.g. obtain and use patient and family feedback* | *-Develop feedback form*  *-Collect feedback*  *-Share feedback* | *-Sarah*  *-All*  *-All* | *3/12* | *Feedback from created and used with 3 patients*  *Meeting attended to discuss* | *If…Sarah forgets to develop form*  *Then…Anna will prompt after 1/12* |
|  |  |  |  |  |  |  |
|  |  |  |  |  |  |  |
|  |  |  |  |  |  |  |

**References**

Michie, S., Atkins, L., & West, R. (2014). The behaviour change wheel. *A guide to designing interventions. 1st ed. Great Britain: Silverback Publishing*, 1003-1010.
