# Supplementary material for: Tailored implementation of a behaviour change intervention for post-stroke physical activity: A mixed-methods feasibility study
Source: Clin Rehabil. 2025 Oct 3;39(12):1589–605. doi: 10.1177/02692155251382502 (PMC12615851; doi:10.1177/02692155251382502)
Supplement: sj-docx-11-cre-10.1177_02692155251382502 - Supplemental material for Tailored implementation of a behaviour change intervention for post-stroke physical activity: A mixed-methods feasibility study [file sj-docx-11-cre-10.1177_02692155251382502.docx]

**Table X Suggested PARAS alterations to enable implementation**

| Improve current PARAS resources | Ability to use whole or parts of the PARAS workbook | *‘…Maybe you almost have a page where there's like tailoring PARAS. So like for inpatients it might be really useful if you could tailor this bit and like your activity sheet and have a resource where you could just download that.’* (Band 7 OT, workshop)  *‘…If we had stuff that we could print off. I guess if it was individual sheets and we could just pick which we might think would be most beneficial for that.’* (Band 6, PT, Focus group)  *‘…And it might be you just want to start the conversation. It might be that you just give them the benefits or barriers just to start with. Then you can give them more.’* (Band 6, PT, Focus group)  *‘…so like a personalised pack rather than just having that one booklet, where you can build your own booklet for them, depending on what they need.’* (Band 6, PT, Focus group)  *‘…The booklets felt really good and they've been able to bring that back to the sessions and we've talked through it as a as a natural resource.’* (Band 6, PT, review)  *‘…One of them had communication difficulties and possibly some cognitive difficulties and that was more of a challenge, certainly for her to do PARAS independently. So we ended up not using the full booklet, but sort of using bits from it that we felt were appropriate for her.’* (Band 7, PT, review) |
| --- | --- | --- |
|  | Offer options of different images on PARAS resources | *‘I found that [invite] useful and maybe hadn't taken that away from when we initially met and talked about the project. So that was something extra that I took from the training.’(Band 6 PT, review)*  ‘…when I sent out the invite, obviously the image at the top is a little bit controversial, but obviously the image is of an elderly person and I just wondered whether there was a possibility to have a younger picture and older picture.’ (Band 6, PT, Review) |
|  | Review outcome measures embedded in PARAS | *‘…I don’t find the IPAQ [International Physical Activity Questionnaire] that helpful.’ (Band 6, PT, review)*  *‘…People just struggled a little bit with it it's like it's quite hard to think about what you've done activity level wise but I think the Diaries are good and I think people do tend to use them.’* (Band 6, PT, focus group)  *‘…Maybe just making it clear that these outcome measures may be helpful for you to use but not necessarily for everyone.’ (Band 6, PT, focus group)*  *‘…So I found it really useful way to have a conversation about honestly, what they were doing when we completed it together. I think is one of my reflections is that having done the IPAQ and having the conversation made the patients reflect usually on how little they were doing. But where I found it difficult was because of the scoring and how hard it is to do. I didn't use it in that way because I just I had to look at the scoring and how to work it out and to be absolutely honest I couldn't make head nor tail of it.’* (Band 7, PT, review)  *‘…I do think it's a good way to open up the conversation and to get them to reflect on what they are or are not doing versus what they think they might be doing.’* (Band 7, PT, review)  *‘…Those two like you say you can get a score and then you can objectively repeat that [The Rivermead Mobility Index] whereas the other one [IPAQ] feels a little bit more subjective you know and yes there was a discussion around it but I didn't think that was necessarily a problem you know I think we still found it useful but it was more that discussion as opposed to a definite increase in hours or increase in time.’* (Band 7, PT, review)  *‘…I was just going to say TOMS* [Therapy Outcome measures] *can feel sometimes a little bit longer whereas you did feel like you could contain it quite quickly [outcome measures included in PARAS] within like 10 minutes of the session.* (Band 6, PT, review) |
|  | Alter physical activity diary to allow measurement of fatigue | *‘…My conversation with the patient and it was just to say that I kind of my session with a patient around fatigue management morphed into PARAS.’* (Band 7, OT, review)  *‘…I actually added a fatigue scale onto the activity diary. So I thought that might be helpful.’* (Band 6, PT, review) |
|  | Improve explanation of physical activity | *‘…I think it was kind of the moving more sitting less just sounded more kind of physical walking rather than using the upper limb, that sort of thing.’* (Band 6, PT, review)  *‘…take out the words physical activity and have it more as a goal setting tool and a treatment planning tool’* (Band 7, OT, focus group) |
|  | Create digital versions of resources to enable clinical notation | *Is there not any online digital versions at all? (Band 7, PT, focus group)*  *‘…So it did feel….a little bit clunky in terms of that documentation. Or quite or a bit more time consuming. So having some sort of booklet online that they could type directly into and e-mail it to you or you could look at it together.’* (Band 7, OT, review)  *‘…she went on and saved the documents into a folder, made them editable for us so that we could email’….So we could e-mail them to patients or we could because so this would be one of the sort of the challenges I suppose was around the documentation around PARAS from our point of view, in terms of our clinical notes.’* (Band 7, PT, review) |
| Develop new PARAS resources | Develop resources to enable use of PARAS within in a group setting | *‘…Do you think it’s something that you could introduce in a group setting so like, on let’s say acute if we brought round about 8 patients that may suitable or maybe with a relative and did it as a group one afternoon.’* (Band 7, PT, focus group)  *‘…I was actually thinking with this it would be quite nice to do it like almost like an education. I'll talk with. Some of the local stroke groups that are ran’* (Band 7 PT workshop)  *‘…I just think it's useful that we have some relatives there because they're the ones that are going to drive them to do it.’* (Band 6, PT, focus group) |
|  | Develop new inpatient resource | *‘…Whether you have like a page that's like more activity and community activities and then a page for the ward’* (Band 7, OT, workshop)  *‘…hospital activities tend to be much more focused around discharge. They tend to be much more focused around like washing and dressing, walking certain distances, transfers etc.’* (Band 7 OT, workshop)  *‘…I don’t know because it is very different in in-patients, isn’t it? They’re at such a different stage. Yeah, it’s difficult to judge that but I think probably separate would be more appropriate*.’ (Band 7, PT, focus group)  *‘…probably less to do might be one that would be more use for the ward and maybe just a bit more ward specific, so maybe not as much information because they’re not going to have as much ability when they’re on a ward situation.’* (Band 6, PT, focus group)  *‘I think the key bits are going through the benefits of exercise, different types of exercise that you can use, thinking about what their goal is, how they’re going to achieve it, the barrier identification and then obviously review it.’ (Band 6, PT, focus group)* |
|  | Adapt PARAS to other conditions | *‘…I can see that it could transfer to other conditions.’* (Band 8a, PT, focus group)  *‘…I think MS in particular there’s quite a few that… like you say, they come in and they want specific exercises for a specific thing but actually I spend a lot of time talking about the benefits of exercising in general and trying to encourage them to do a little bit more but then it’s that monitoring of that and linking that with the fatigue and I think having goals and things that they can work towards. I think that would definitely help*. (Band 4, therapy assistant, focus group)  *‘…When I was choosing patient that this is not on that this is for the strokes because for me it sits well with and with other conditions as well.’* (Band 6 PT, review)  *‘…Anything, really, when you start to talk about activity and exercise, you do find yourself talking about it in a PARAS way with someone who's not a stroke.’* (Band 7, PT, review)  *‘…I think it could be used with other other conditions.’* (Band 7, OT, review) |
